# Glyphosate Shapes a Dinoflagellate-Associated Bacterial Community While Supporting Algal Growth as Sole Phosphorus Source

**DOI:** 10.3389/fmicb.2017.02530

**Published:** 2017-12-19

**Authors:** Cong Wang, Xin Lin, Ling Li, LingXiao Lin, Senjie Lin

**Affiliations:** ^1^State Key Laboratory of Marine Environmental Science, College of Ocean and Earth Sciences, Xiamen University, Xiamen, China; ^2^Department of Marine Sciences, University of Connecticut, Groton, CT, United States

**Keywords:** glyphosate, P-source, *Prorocentrum donghaiense*, microbial community, 16s rRNA gene profiling, *phnJ*

## Abstract

Glyphosate is a widely used herbicide that can potentially be a phosphorus (P) source for phytoplankton and microbes when discharged into the coastal ocean. In contrast to bacteria, few eukaryotic phytoplankton species appear capable of directly utilizing glyphosate. In this study, we observed, after a long delay (>60 days), *Prorocentrum donghaiense*, a dinoflagellate known to cause major harmful algal blooms in the East China Sea, could grow in a medium with glyphosate as the sole P source; suggesting that *P. donghaiense* growth was through bacterial mediation. To understand how the bacteria community might respond to glyphosate, we analyzed the 16S rRNA genes of the microbial community present in *P. donghaiense* cultures when grown under lower (36 μM) and higher (360 μM) glyphosate concentrations. Based on both Sanger and Illumina high throughput sequencing, we obtained more than 55,323 good-quality sequences, which were classified into six phyla. As the concentration of glyphosate rose, our results showed a significant increase in the phyla Proteobacteria and Firmicutes and a decrease in the phylum Bacteroidetes. Further qPCR (Quantitative PCR) analysis showed higher abundances of two specific phylotypes in the higher-glyphosate *P. donghaiense* cultures when compared to the lower-glyphosate and no-glyphosate cultures. Correspondingly, qPCR displayed the same trend for the abundance of a gammaproteobacterial type of *phnJ*, a gene encoding Alpha-D-ribose 1-methylphosphonate 5-phosphate C-P lyase, which is responsible for phosphonate degradation. In addition, Tax4Fun analysis based on our 16S rRNA gene sequences results in higher predicted abundances of phosphonate metabolizing genes in glyphosate-treated cultures. This study demonstrates that glyphosate could selectively promote the growth of particular groups of bacteria within an algal culture and in glyphosate enriched coastal waters, this interaction may potentially further facilitate the growth of alga.

## Introduction

Glyphosate [N-(phosphonomethyl) glycine] is a broad spectrum, post-emergent, herbicide widely used in agriculture and silviculture to inhibit the growth of grasses. It works through the inhibition of 5-enolpyruvyl shikimic acid-3-phosphate (EPSP) synthase and disrupts aromatic amino acid biosynthesis (Duke, 2003). The inhibition decreases or halts protein synthesis, causing cessation of growth, cell disruption, and death of the impacted grasses (Ali and Fletcher, 1978; Hernando et al., 1989; Schaffer and Sebetich, 2004). Glyphosate is extensively used worldwide, topping lists of agricultural herbicide usage in Europe (Nadin, 2007) and the U.S. (Grube et al., 2011). Glyphosate infiltrated into the soil is subjected to degradation by the microbial community; however, pulses of coastal water contamination can be expected when rainfall occurs directly after application and when flood events increase river sediment load (Giesy et al., 2000). Urban runoff and wastewater treatment effluent also account for considerable glyphosate inputs into rivers (Botta et al., 2009). Reports showed that glyphosate was detected in more than 50% of 3,732 environmental water and sediment samples collected between 2001 and 2010 in the U.S. with a median glyphosate concentrations of < 0.02 μg/L (0.12 nM) and a maximum of 476 μg/L (2.86 μM; Battaglin et al., 2014). Moreover, considerably higher concentrations have been detected under direct aquatic application and in isolated wetland environments (Giesy et al., 2000; Battaglin et al., 2009).

Glyphosate has been considered environmentally safe because no harmful effects are expected on humans and other animals, as they do not possess the shikimate pathway (Giesy et al., 2000; Duke and Powles, 2008). However, bacterial communities in aquatic ecosystems have several reports of diverse biological effects (Rohr and Crumrine, 2005; Pérez et al., 2007; Pesce et al., 2009). Furthermore, studies have shown that some strains of bacteria and marine algae can utilize glyphosate as a source of P nutrient (Hove-Jensen et al., 2014; Wang et al., 2016), while others are unable to utilize or are even inhibited by glyphosate (Forlani et al., 2008; Wang et al., 2016). This variability may have to do with whether these microbes have a phosphonate hydrolyzing enzyme system or the shikimate pathway. This raises a question whether algal-bacterial associations (Caldwell and Overbeck, 1977) can play a role in glyphosate P scavenging. Many symbiotic associations between algae and bacteria provide the algae with a supply of vitamins (Menzel and Spaeth, 1962; Haines and Guillard, 1974; Swift and Guilford, 1978; Croft et al., 2005), phosphorus and/or nitrogen (Golterman, 1972; Bloesch et al., 1977; Axler et al., 1981), or growth regulators (Bunt, 1961; Delucca and McCracken, 1977) and in return, algae provide photosynthetically fixed carbon to bacteria (Overbeck and Babenzien, 1964; Anderson and MacFadyen, 1975; Hobbie and Rublee, 1977). However, what type of bacteria can mediate the accessibility of phosphorus in glyphosate to their partner algae is poorly understood.

*Prorocentrum donghaiense* is a dinoflagellate that regularly forms spring blooms in the East China Sea. *P. donghaiense* is known to be capable of survival in P-limited environments through the utilization of DOP to support its growth. In this study, we found that *P. donghaiense* was unable to directly utilize glyphosate as a sole P-source; however, after a long delay (likely due to induction of bacterial growth), *P. donghaiense* was able to grow on glyphosate. We set up experiments with different glyphosate treatments to examine how glyphosate may impact the bacterial community in general and which lineages in the *P. donghaiense* cultures contain glyphosate-hydrolyzing enzymes. The results were used to test two hypotheses: (1) glyphosate can cause a shift in the bacterial community present in the *P. donghaiense* culture and (2) the shift would involve an increase in glyphosate-degrading species. The verification of both these hypotheses would lead to the conclusion that the glyphosate-induced bacteria can mediate the release of DIP from glyphosate to support the growth of *P. donghaiense*.

## Materials and methods

### Algal cultures

*Prorocentrum donghaiense* was kindly provided by the Collection Center of Marine Algae, Xiamen University, China. All the cultures were aseptically handled and grown in autoclaved and 0.22 μm filtrated seawater with f/2 -silicate medium (Guillard, 1975). Seawater used in this study was collected from the South China Sea and its background DIP concentration was measured (see below for the measurement method) and found to be low (0.73 μM). Cultures were maintained at 20°C under a light: dark cycle of 14:10 h with a photon flux of 100 μmol·m^−2^·s^−1^.

### Experimental design

Stock solution of pure glyphosate (99.9%, non-derived compound; SIGMA -ALDRICH) was prepared at a final concentration of 36 mM, sterilized by filtration through 0.22-μm membranes, and stored at 4°C. *P. donghaiense* cultures were set up in five different P nutrient conditions (f/2, f/2^−P^, f/2^−P+Gly^, f/2^LG^, and f/2^HG^). Due to the lack of an accurate method for glyphosate measurement in coastal waters, it is difficult to know the glyphosate distribution in the ocean. Thus, in this paper, 36 μM glyphosate (group f/2^−P+Gly^) was used, a concentration equivalent to the phosphate concentration in the f/2 medium, as the sole P-source for comparison with the DIP-depleted cultures, f/2^−P^ (DIP concentration was <1 μM). Furthermore, 36 μM (f/2^LG^) and 360 μM (f/2^HG^) glyphosate were added to the normal (f/2) media to test for toxic effects. A high concentration of glyphosate was used because: (1) we wanted to maximize the chance to elicit a response of the bacterial community in the *P. donghaiense* culture and (2) these concentrations were used in our previous study in which 360 μM glyphosate was found to cause severe inhibition for most phytoplankton species (Wang et al., 2016), except *P. donghaiense*. Each treatment was set up in triplicate and all experiments were run in a volume of 400 mL in 600 mL Cell and Tissue Culture Flasks (Jet Biofil). Because dinoflagellates do not grow well under mechanical disturbance, the cultures were not agitated but were mixed gently whenever a sample was taken. At the end of the experiment (day 10), a slight increase in cell concentration was observed in the last two media (LG and HG). To further investigate the effects of glyphosate on these cultures, f/2^LG^ and f/2^HG^ were extended to 122 days.

### Growth monitoring and measurement of parameters

Experiments with glyphosate began when the stock cultures entered the exponential growth stage. For all five P-nutrient conditions described above, the initial cell concentration of *P. donghaiense* was set to ~10% of the highest concentration, or 8,000 cells/mL, to provide room for growth. To monitor growth, 1 mL culture was removed to perform a cell count every 2–3 days using a Sedgwick-Rafter counting chamber under the microscope (LeGresley and McDermott, 2010). Meanwhile, DIP was measured using the molybdate-ascorbic method by filtrating 25 mL of culture (Parsons, 2013) and the detection limit of the machine was 0.01 μM. These measurements were carried out for a period of 10 days, until growth of all treatments reached a plateau except for the glyphosate-treated cultures, which showed no significant growth throughout. After day 10, the cultures were checked approximately every 2 weeks. On day 122, remarkable growth was observed in the cultures with glyphosate and concentrations of DIP or DIN (dissolved inorganic nitrogen) were again determined.

### DNA extraction, amplification of the 16S rRNA gene, and sequencing

On day 122, about 25 mL of culture was collected from each treatment and was filtrated by using a sterilized 0.22-μm nitrocellulose membrane. The membrane was then transferred into a 1.5 mL microcentrifuge tube with 0.8 ml of DNA extraction buffer (containing 0.1 M EDTA, 1% sodium dodecyl sulfate, 8 μg lysozyme, and 18 μg proteinase K). The samples were incubated at 55°C for 3 days, with the prolonged incubation intended to maximize cell breakage. DNA extraction was then carried out using a CTAB protocol combined with the Zymo DNA Clean & Concentrator kit (Zymo Research Corp., Orange, CA), as previously reported (Zhang et al., 2005).

From each sample, 1 μL (~80 ng) of the extracted DNA was used as the PCR template to amplify the full-length 16S rRNA gene. Amplification was run in the following program; initial denaturation at 95°C for 3 min, followed by 35 cycles of denaturation at 94°C (30 s), annealing at 56°C (30 s) and extension at 72°C (1 min), with a final extension cycle at 72°C for 10 min. Degenerate primers 27F [5′-AGAGTTTGATCMTGGCTCAG-3′ (M is A or C)] and sgR (5′-TAGGGTTACCTTGTTACGACTT-3′) were used as they were previously found to amplify approximately a 1.5-Kb fragment of 16S rRNA in a wide range of bacteria (Webster et al., 2003; Xu et al., 2011; Zhou et al., 2014). The amplicons were gel purified and cloned. The resulting clones were randomly picked from each treatment for Sanger sequencing.

Although Sanger sequencing provides long reads (~800 bp) and hence higher taxonomic resolving power, its data output from a feasible sequencing scale is limited and as such, limits the depth coverage of existing taxonomic diversity. To address this issue, we also conducted Illumina high-throughput sequencing. V4-V5 specific primers, 515F (forward primer, 5′-GTGCCAGCMGCCGCGG-3′) and 907R (reverse primer, 5′-CCGTCAATTCMTTTRAGTTT-3′), were used to amplify a 390-bp 16S rRNA gene fragment from the DNA template. PCR was carried out as the following: 95°C for 2 min, followed by 30 cycles of incubation at 95°C (30 s), 55°C (30 s) and 72°C (30 s), with a final extension cycle of 5 min at 72°C. The amplicons obtained through three independent PCRs for each of the DNA template preparations were purified and subjected to the Illumina Miseq (Majorbio Co. Ltd., Shanghai, China).

### Sequences data analysis

Nucleotide sequences acquired through Sanger sequencing were subjected to a chimera check using the Bellerophon v.3 program (Huber et al., 2004; http://greengenes.lbl.gov) with default settings. Sequences flagged as potential chimeras were examined further by comparing the BLAST results of both the sequences flanking the breakpoint with suspected parental sequences. If both the parental sequences were from the same PCR reaction of our own sample, we ranked the sequence as a potential chimera. Next, the end sequences of the same potential chimeric sequence were BLASTed against GenBank database separately. If the two fragments were highly identical (>97%) to different species of bacteria, the sequence was ranked as a chimera. After the removal of the chimera sequences, the remaining sequences were aligned using MUSCLE on MEGA (Hall, 2013) and the criteria to define an operational taxonomic units (OTUs) was set as 97% similarity.

All the sequences obtained from MiSeq are available in the Sequence Read Archive (SRA, http://www.ncbi.nlm.nih.gov/Traces/sra). We used the Trimmomatic (Bolger et al., 2014) and FLASH (Magoč and Salzberg, 2011) software packages to conduct quality filtering of raw reads. Reads with trailing bases of below quality 20 (Phred Quality Score) were trimmed and a sliding window of 50 bp was used to trim tail reads that showed an average quality within the window < 20. Reads shorter than 50 bp were discarded. Setting the minimum overlap length as 10 bp and maximum mismatch ratio as 0.2, pair-end reads were assembled into contigs. These contigs were then assigned to treatment groups according to barcode with maximum allowed mismatch of 2 bases.

The contigs were clustered at 97% identity cutoff (generally considered to define microbial species) into OTUs to calculate rarefaction and non-parametric estimators (such Chao1 and ACE) using UPARSE (version 7.1) (Edgar, 2013). Chimeras were detected and removed using the UCHIME pipeline (Edgar et al., 2011). Taxonomic assignments of the OTUs were performed using the RDP classifier (version 2.2) (Wang et al., 2007). The SILVA database (release 115) (Quast et al., 2012) was used for the retraining of RDP classifier and the minimum confidence threshold was set to 0.70. Only five sequences in the V4-V5 library remained unclassified below the domain Bacteria (Table 1A).

**Table 1:** Sequencing characteristics, distributions and classification.

**A. TOTAL SEQUENCES**			
	V4-V5		
Total sequences	53,609		
Classified bacteria	53,604		
Unclassified bacteria	5		
**B. TOTAL SEQUENCES IN EACH SAMPLE**			
	V4-V5		
C	17,221		
LG	11,601		
HG	24,787		
**C. TOTAL SEQUENCES CLASSIFICATION**			
Phylum/Class	C	LG	HG
Proteobacteria	6,774 (39.3%)	6,436 (55.48%)	15,917 (64.22%)
Alphaproteobacteria	4,278	4,532	10,729
Betaproteobacteria	157	149	5
Deltaproteobacteria	162	521	3,294
Gammaproteobacteria	2,177	1,234	1,889
Bacteroidetes	9,924 (57.63%)	4,406 (37.98%)	7,977 (32.18%)
Cytophagia	5,577	3,068	6,638
Sphingobacteria	3,893	1,038	202
Flavobacteria	5	48	147
Bacteroidia	2	3	0
Actinobacteria	16 (0.09%)	5 (0.04%)	11 (0.04%)
Actinobacteria	4	3	7
Clostridia	2	3	5
Thermolephilia	12	2	4
Firmicutes	4 (0.02%)	4 (0.03%)	12 (0.05%)
Bacilli	1	1	6
Negetivicutes	1	0	1
Planctomycetes	489 (2.84%)	750 (6.46%)	865 (3.49%)
Phycosphaerae	489	750	865
WCHB1-60	13 (0.08%)	0 (0.00%)	1 (0.00%)

For the prediction of functional profiles of the bacterial community based on our 16S rRNA gene sequences, data from each treatment and the control were run through the newly developed open-source R package Tax4Fun (Aßhauer et al., 2015) after being processed with Qiime (based on SILVA).

### qPCR to quantify target phylotypes and glyphosate metabolizing gene *phnJ*

Copy numbers of two phylotypes (16S rRNA gene) and *phnJ* were determined using qPCR (Quantitative PCR), which was conducted on the CFX96 Real-Time System (Bio-Rad, USA). The two 16S rRNA gene phylotypes belonged to Actinobacteria and Gammaproteobacteria, which were not detected in the control but had high copy numbers in the LG or HG groups. Specific primers for the 16S rRNA genes of these two phylotypes were designed based on the sequences we obtained. For *phnJ*, we first used degenerate primers (PhnJF1 and PhnJR1; Table 2) that were based on the alignment of thirteen *phnJ* sequences from GenBank's Gammaproteobacteria to identify conserved regions of this gene (Figure S2). Using these primers in PCR, we obtained a gene fragment of about 340 bp and sequenced 10 clones which revealed that all the eight sequences (Figure S3) we obtained from the colonies hit the *phnJ* gene in the database (the other 2 were not a resolved single sequence and were discarded). Based on these sequences, we designed several sets of primers for qPCR. The specificity of these primers was tested using qPCR melt curve analysis. One of the primer pairs was found to amplify one single product (melt curve showing single peak; Figure S4) and was selected for use in qPCR (PhnJF2 and PhnJR2; Table 2). The PCR products were purified and prepared in successive 10-fold dilutions to be used as standards. The same molar quantity of genomic DNA (5 ng) was used as the template to amplify the target genes from each sample group (C, LG, and HG). Their copy numbers were calculated based on the standards, which were amplified on the same PCR runs as the samples. Each PCR reaction was carried out in a total volume of 12 μL containing 6 μL of 2 × iQSYBR Green supermix (Bio-Rad, USA), 375 nM of each primer, and 6 μL of 5 ng/μL DNA. The PCR program was composed of a denaturation step of 3 min at 95°C, followed by 40 cycles of 95°C for 10 s and 60°C for 32 s. In each run, negative controls were set up without DNA templates. Each reaction had three technical replicates. At the end, to confirm primer specificity, all the PCR products were subjected to melting curve analysis.

**Table 2:** The sequences and annealing temperatures (Ta's) of the specific primers used in QPCR.

**Primers**	**Sequences(5′-3′)**	**T_a_(°C)**
Phylotype1F	GGCAGACTAGAGTGTGGTA	58
Phylotype1R	TGAGACCCACACCTAGTTC	58
Phylotype2F	TGGCAGACTAGAGTCTTG	54
Phylotype2R	CTACGCACGCCTTAAAGG	56
phnJF1	ATGCCRMTGCCBTAYGGYTGGGMACCGGYGGYAT	72
phnJR1	TGCATWACWCCGTABTCNTCWAGCGCRTGCAT	66
phnJF2	GATCGGCACCTGGTAGACCA	56
phnJR2	GGCTCAAGGTCATCGATCA	51

### Statistical analysis

For all variables, differences among treatments were examined using repeated measure analysis of variance (RM ANOVA; Winer et al., 1971), with three glyphosate treatments (f/2, f/2^−P^, f/2^−P+Gly^ and f/2, f/2^LG^, f/2^HG^, separately) and sampling times as treatment matrices. RM ANOVA analyses were followed by all pairwise multiple comparisons (post hoc testing), using the Holm–Sidak method (*P* < 0.05).

## Results

### Effects of glyphosate on growth of *P. donghaiense*

**Figure 1:**
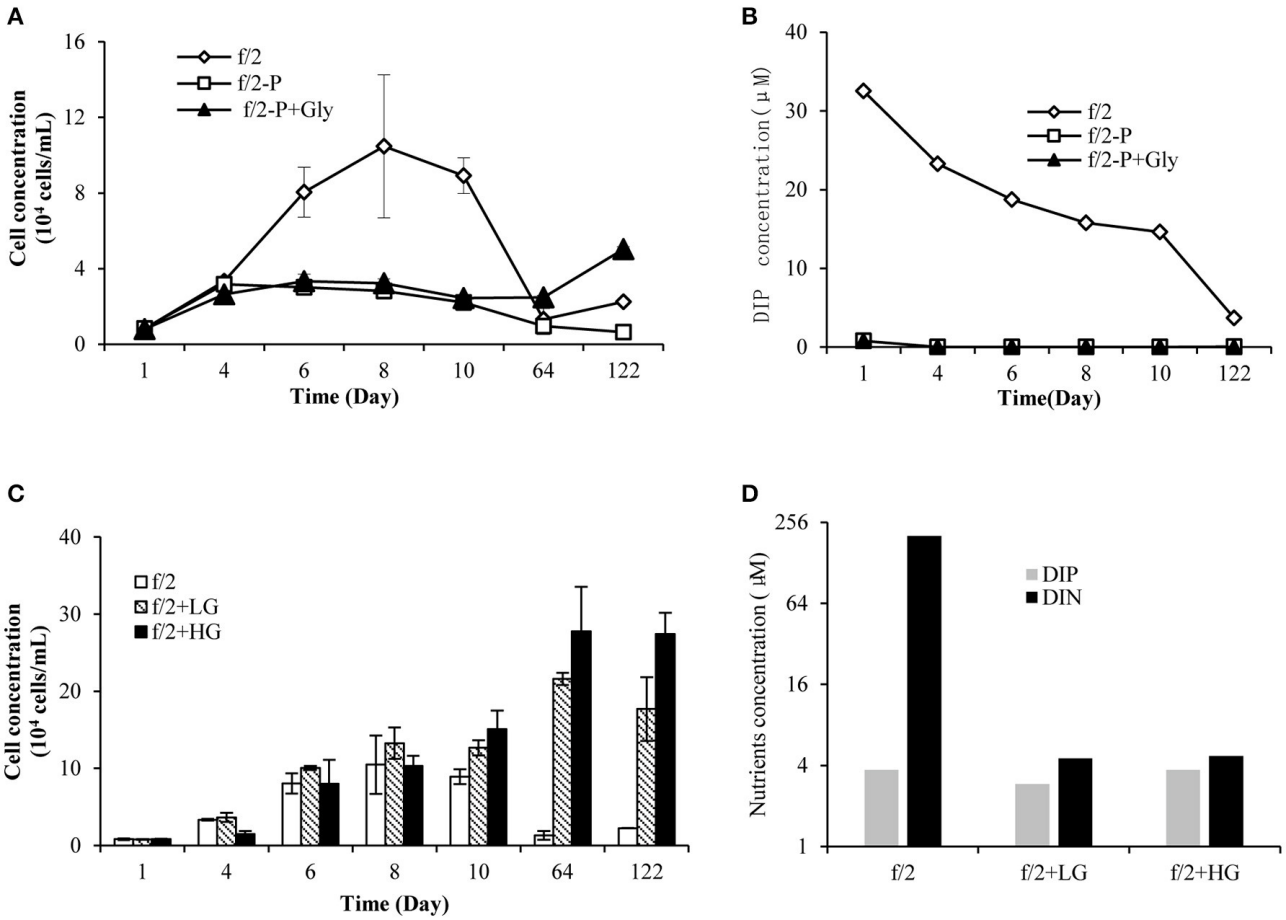
Growth and nutrient conditions of *P. donghaiense* in all experimental settings used in this study. **(A)** Growth of *P. donghaiense* in cultures f/2, f/2^−P^, and f/2^−P+Gly^; **(B)** DIP concentrations in cultures f/2, f/2^−P^, and f/2^−P+Gly^; **(C)** Growth of *P. donghaiense* in cultures f/2, f/2^LG^, and f/2^HG^; **(D)** DIP and DIN concentrations in cultures f/2, f/2^LG^, and f/2^HG^ on day 122.

Starting with equal cell concentrations, the f/2-group grew exponentially and peaked on day 8, while the f/2^−P^ and f/2^−P+Gly^ treatments entered the plateau phase on day 4 (Figure 1A). Statistical analysis showed that there was a significant difference in growth between groups f/2 and f/2^−P^ (*p* < 0.05, RM ANOVA) from day 1 to day 10, while no significant difference was observed between f/2^−P+Gly^ and f/2^−P^ (*p* > 0.05, RM ANOVA). Meanwhile, DIP concentrations in the f/2 cultures decreased steadily whilst those in the f/2^−P^ and f/2^−P+Gly^ treatments were low to begin with (limited to the carry-over from the culture inoculum or seawater) and further dropped to below the detection limit from day 4 (Figure 1B). These results indicated that *P. donghaiense* could not hydrolyze glyphosate to obtain phosphate. On day 64, the f/2 and f/2^−P^ almost died due to depletion of available P-source whereas the f/2^−P+Gly^ group could still maintain slight growth (Figure 1A), showing a positive albeit delayed effect caused by glyphosate. Furthermore, on day 122, significant increasing growth was observed in f/2^−P+Gly^ compared to f/2 and f/2^−P^. However, DIP in f/2^−P^ and f/2^−P+Gly^ cultures was still below the detection limit on this day. The late-stage increased growth in f/2^−P+Gly^ might be because glyphosate induced bacteria in the culture to degrade glyphosate and release phosphate, which was taken up immediately by *P. donghaiense*. We also noticed that as early as day 10, the cell concentrations of both the f/2^LG^ and f/2^HG^ groups showed higher (1.4- and 1.7-folds, respectively) than that in the f/2 group (Figure 1C). On day 64 and 122, when the f/2 cultures declined dramatically due to nutrient depletion, groups f/2^LG^ and f/2^HG^ exhibited a bloom equivalent condition, with a higher biomass in f/2^HG^, and this trend lasted for at least 2 months (Figure 1C). Correspondingly, the color of cultures became increasingly darker with increasing concentrations of glyphosate among these three groups (pictures not shown). And on day 122, DIP concentrations in all three groups were < 4 μM, indicating that DIP was nearly depleted. DIN in f/2^LG^ and f/2^HG^ was also depleted (4.5 and 4.7 μM, respectively) compared to the abundant presence in f/2 (203 μM; Figure 1D), indicating further nitrogen consumption and growth in the glyphosate-based cultures, beyond what f/2 cultures could achieve.

### Bacterial diversity analysis from 16S rRNA gene clone libraries

**Figure 2:**
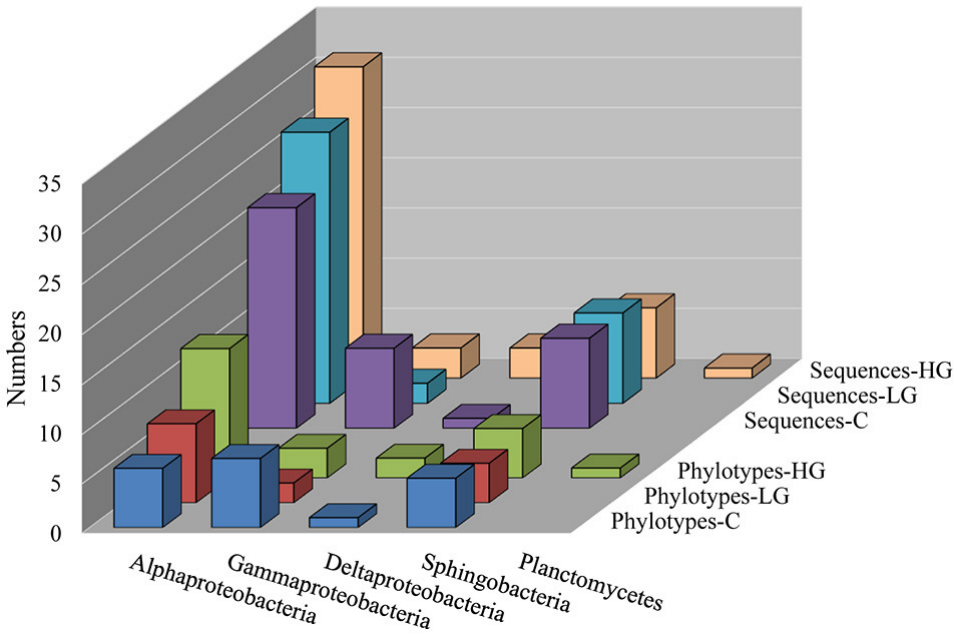
Effects of glyphosate on bacterial community in *P. donghaiense* cultures. The bacterial richness (numbers of phylotypes) and abundance (numbers of sequences) in five main groups Alphaproteobacteria, Gammaproteobacteria, Deltaproteobacteria, Sphingobacteria, and Planctomycetes in cultures amended with different concentrations of glyphosate. C, the control, f/2 medium; LG, f/2 medium amended with lower concentration (36 μM) of glyphosate; HG, f/2 medium amended with higher concentration of glyphosate (360 μM).

Bacterial communities in the C, LG, and HG cultures were compared on day 122 based on 16S rRNA gene. We randomly picked and sequenced 150 clones derived from 16S rRNA gene amplicons. After excluding 27 sequences because of evidence of sequencing error or likely being chimeric DNA, the remaining good-quality sequences were classified into different phylotypes to characterize changes in major functional groups among C, LG, and HG (Figure 2). It is clear that the microbial community was composed of Proteobacteria (in declining richness order from Alphaproteobacteria, Gammaproteobacteria to Deltaproteobacteria), Bacteroidetes (Class Sphingobacteria), and Planctomycetes.

Results showed that there were 17 phylotypes identified in the control, while 9 and 22 phylotypes in LG and HG respectively. A similar trend was found for Shannon–Wiener diversity index, a commonly used diversity index in ecological literature (Spellerberg, 2008), which was 2.28, 1.80, and 2.71 for C, LG, and HG, respectively. This result indicates that 36 μM glyphosate caused a decrease in the bacterial diversity but 360 μM glyphosate caused an increase. Of the bacteria detected, Alphaproteobacteria were dominant and their richness and abundance went up along with the increase in glyphosate concentration. As for Gammaproteobacteria, a higher richness was observed in C in comparison to LG and HG, displaying a depressed effect of glyphosate on this group. By contrast, we identified Deltaproteobacteria only in C and HG. No significant difference was observed in richness and abundance among group C, LG, and HG for Sphingobacteria, showing that this might be a core group of bacteria constitutively associated with *P. donghaiense*. Only one phylotype was identified in Planctomycetes and it only occurred in HG.

### Bacterial diversity analysis from 16S rRNA gene high-throughput sequencing data

More than 53,000 16S rRNA gene V4-V5 sequences (Table 1A) were obtained using Illumina high-throughput sequencing. Most of the curated sequences (exceeding 99.9%) were classified to a particular bacterial phylum and class (or lower taxon). Sequences that were unclassified below the level of Bacteria probably represented a mixture of true biodiversity of novel taxa and complex chimeras that were not identified by UCHIME pipeline (see section Materials and Methods). In this dataset, different read numbers were obtained in groups C, LG, and HG (Table 1B), but the rarefaction curves were nearly asymptotic (Schopf, 1975) for C and LG, while our sampling for HG was less but still nearly complete (Figure S1).

#### Overall bacterial composition

Sequence classification showed that Proteobacteria and Bacteroidetes were the dominant phyla, together representing more than 90% in each of the glyphosate treatment groups (Table 1C). Planctomycetes constituted 2.84% in group C (~500 sequences) but the percentage doubled after being induced by glyphosate, which showed much more information than that in the 16S rRNA gene clone libraries where only one sequence was identified in the HG group. Actinobacteria, Firmicutes, and WCHB1-60 together accounted for < 0.1%. The abundance of Actinobacteria and WCHB1-60 dropped along with the increasing concentration of glyphosate, but on the contrary the abundance of Firmicutes went up.

#### Differential response of different phylotypes to glyphosate

**Figure 3:**
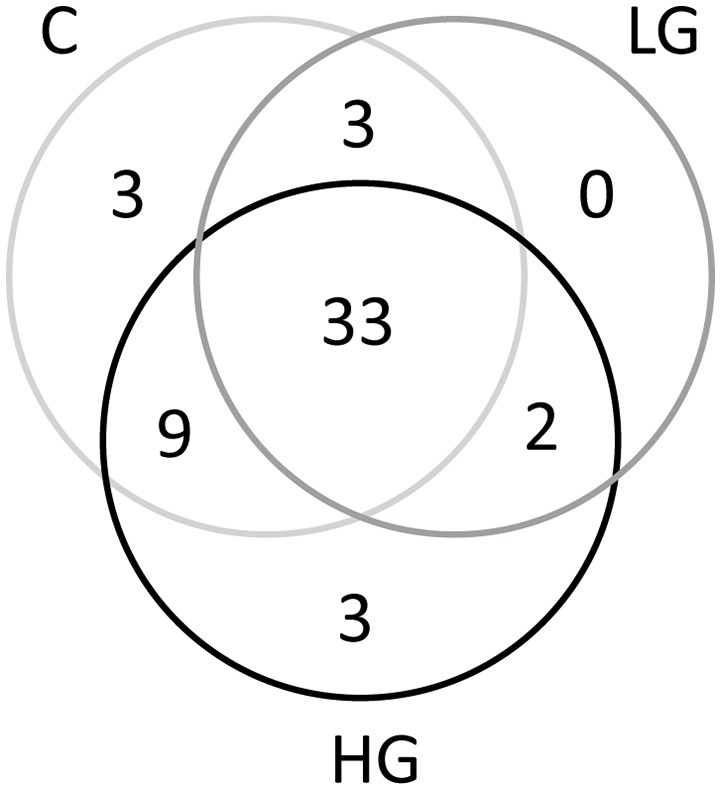
Venn diagrams (at distance 0.03) showing the shared as well as specific phylotypes in groups C, LG, and HG.

**Figure 4:**
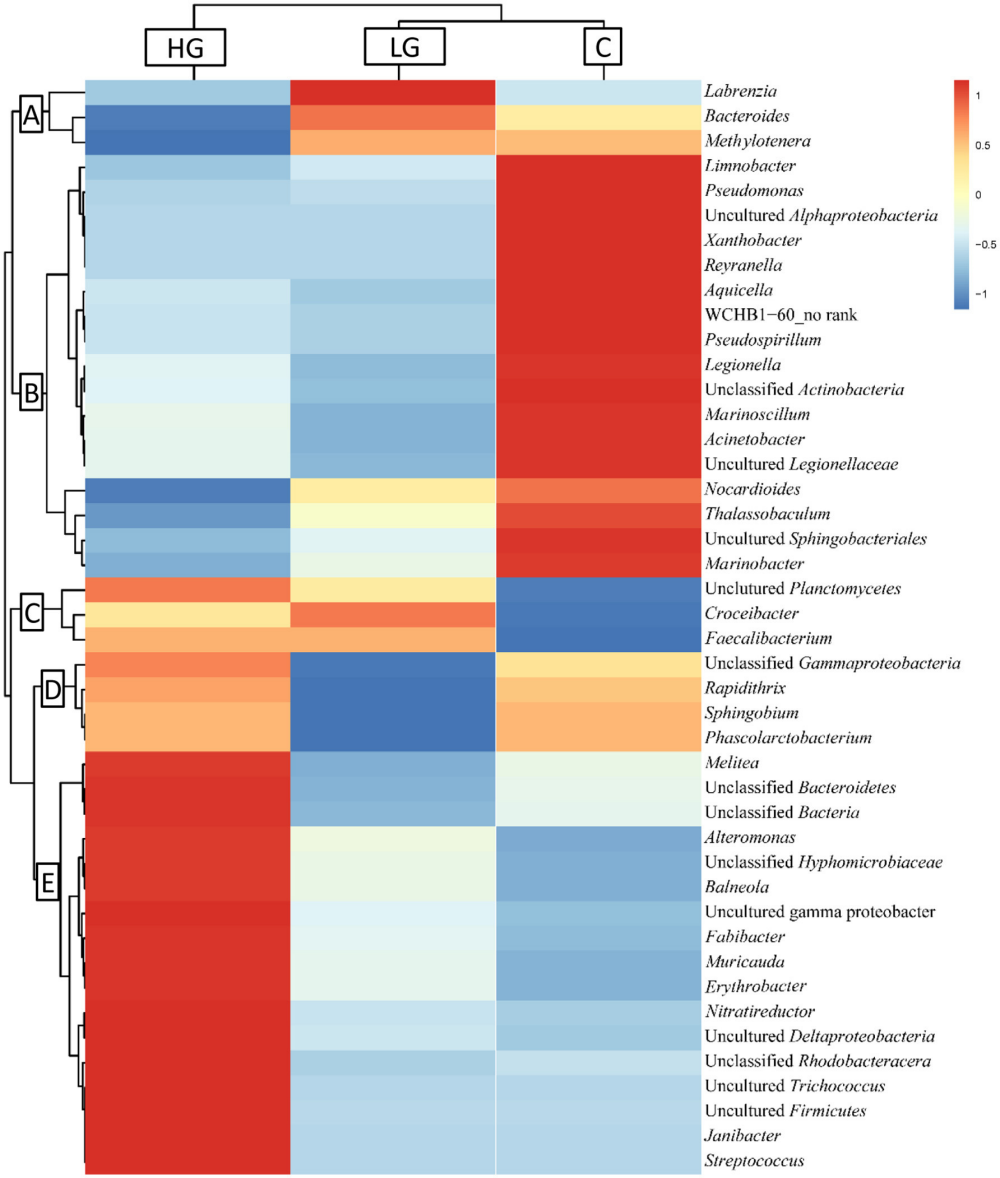
Hierarchical clustering of V4-V5 region of 16S rRNA gene based on microbiota compositions. Forty-four phylotypes were classified as differentially abundant and grouped by similarity of patterns. The spectral percentage for each phylotype was averaged across treatments (C, LG, and HG). Red color indicates increased abundance while blue indicates reduced abundance. Letters (A-E) indicate clusters of similar pattern.

After blast analysis was performed to classify the sequences to the genus level, a total of 53 phylotypes were identified. The bacterial composition in group C, LG, and HG was highly similar and they shared 33 phylotypes (Figure 3), while only 3 phylotypes were specific to HG. These phylotypes were then hierarchically clustered to categorize sequences according to their abundances and these sequences were further cataloged to five distinct clusters across the three treatments (A–E; Figure 4).

Three phylotypes were most abundant under LG conditions, which fell into cluster A. The richness of these phylotypes was promoted in 36 μM glyphosate but decreased in 360 μM glyphosate. For instance, the richness of *Labrenzia* spp. in the LG group was about 10-fold that of the control group, but it dropped to zero in the HG group, suggesting that *Labrenzia* spp. have a strong positive response to 36 μM glyphosate but did poorly under 360 μM glyphosate. Cluster B included 17 phylotypes whose abundances were highest in the control. Most of the blast hits of these phylotypes were aquatic microbes. The most abundant phylotype detected was *Marinoscillum* spp., which was down-regulated by 2-fold in the HG treatment when compared to the control. In this cluster, we even only detected from fewer than 10 to no sequences in the LG or HG groups for some bacteria such as *Pseudomonas* spp., *Legionella* spp., *Aquarelle* spp., *Xanthobacter* spp., and *Marinobacter* spp. Cluster C included 3 phylotypes, whose abundances were the lowest in the control, but elevated dramatically in the presence of glyphosate. *Croceibacter* spp., in particular, was not detected in the control and only identified in the cultures with glyphosate. Phylotypes in cluster D were least abundant in LG, but in HG their abundances increased to nearly the same as in the control. Seventeen phylotypes that were most abundant in HG were grouped in cluster E, most of which also showed slightly higher abundance in LG relative to the control. The most abundant phylotype in this cluster was *Fabibacter* spp., constituting 0.37% in the control, 4.01% in LG, and 7.23% in HG treatments.

#### qPCR quantification of copy numbers for target genes and Tax4Fun community functional prediction

**Figure 5:**
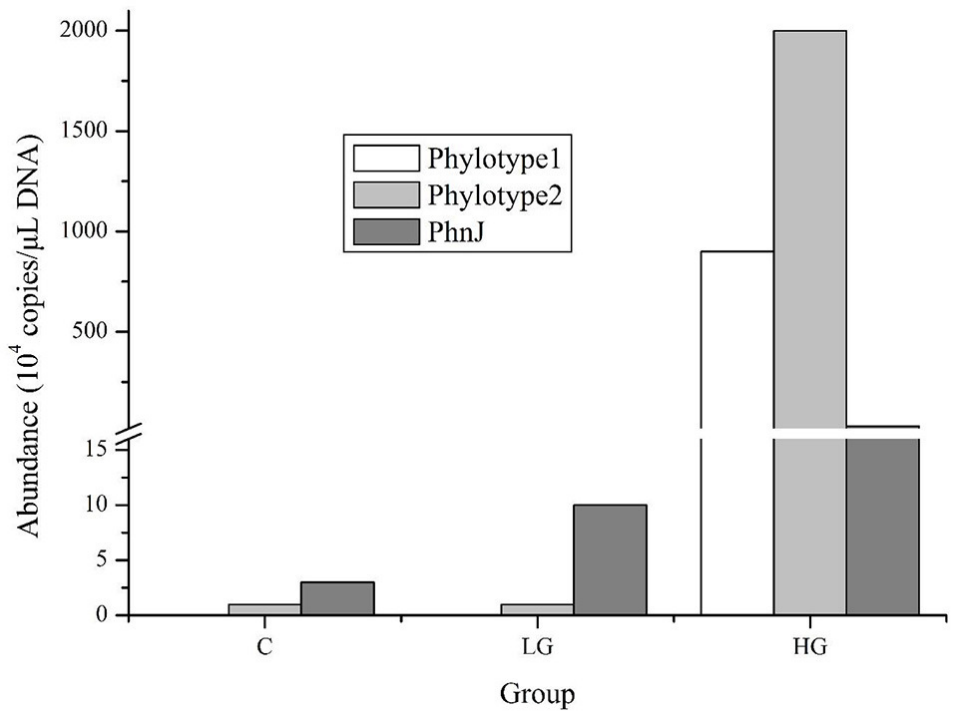
Abundance of two phylotype 16S rRNA genes and *phnJ* gene in different glyphosate treatment groups. In high-throughput sequencing libraries, phylotype 1 was only detected in group HG while phylotype 2 was detected in both LG and HG groups and its abundance increased orderly. The qPCR data for *phnJ* gene was generated from primers phnJF2 and phnJR2 shown in Table 2.

We conducted qPCR for two representative phylotypes belonging to cluster E, whose abundances were higher in LG or HG than in the control in the high-throughput sequence data, and for the gene *phnJ*, the core gene required for glyphosate degradation. The two phylotypes showed higher copies in HG by more than 60,000- and 2,000-fold when compared to the control and LG treatments, respectively (Figure 5). Correspondingly, the copy number of *phnJ* that was amplified by our primers also showed an upward trend from 30,000 in the control to 100,000 in LG and 300,000 in HG.

From the results of Tax4Fun analysis, we sorted out genes related to phosphonate uptake and metabolism from each of our samples and calculated the ratio of Tax4Fun values from a treatment to the control. We found that the ratios of LG/C and HG/C were all >1 and the latter always exceeded the former with only one exception (Table S1). This result indicates that the bacterial communities' phosphonate metabolism gene abundance increased as glyphosate concentrations went from 0 to low and high concentrations. We noticed that the percentage of OTUs that were actually mapped to KEGG organisms for C, LG, and HG was 75.8, 70.8, and 56.4%, respectively. This means that Tax4Fun predicted abundances of phosphonate-metabolizing genes might even be underestimates, particularly for LG and HG groups.

#### Glyphosate shaped the bacterial community structure in each group

In the high-throughput dataset, the Shannon–Wiener diversity index based on OTU was 2.23, 2.62, and 2.6 for the control, LG, and HG community, respectively, suggesting that glyphosate exposure increased the microbial community diversity in *P. donghaiense* cultures.

**Figure 6:**
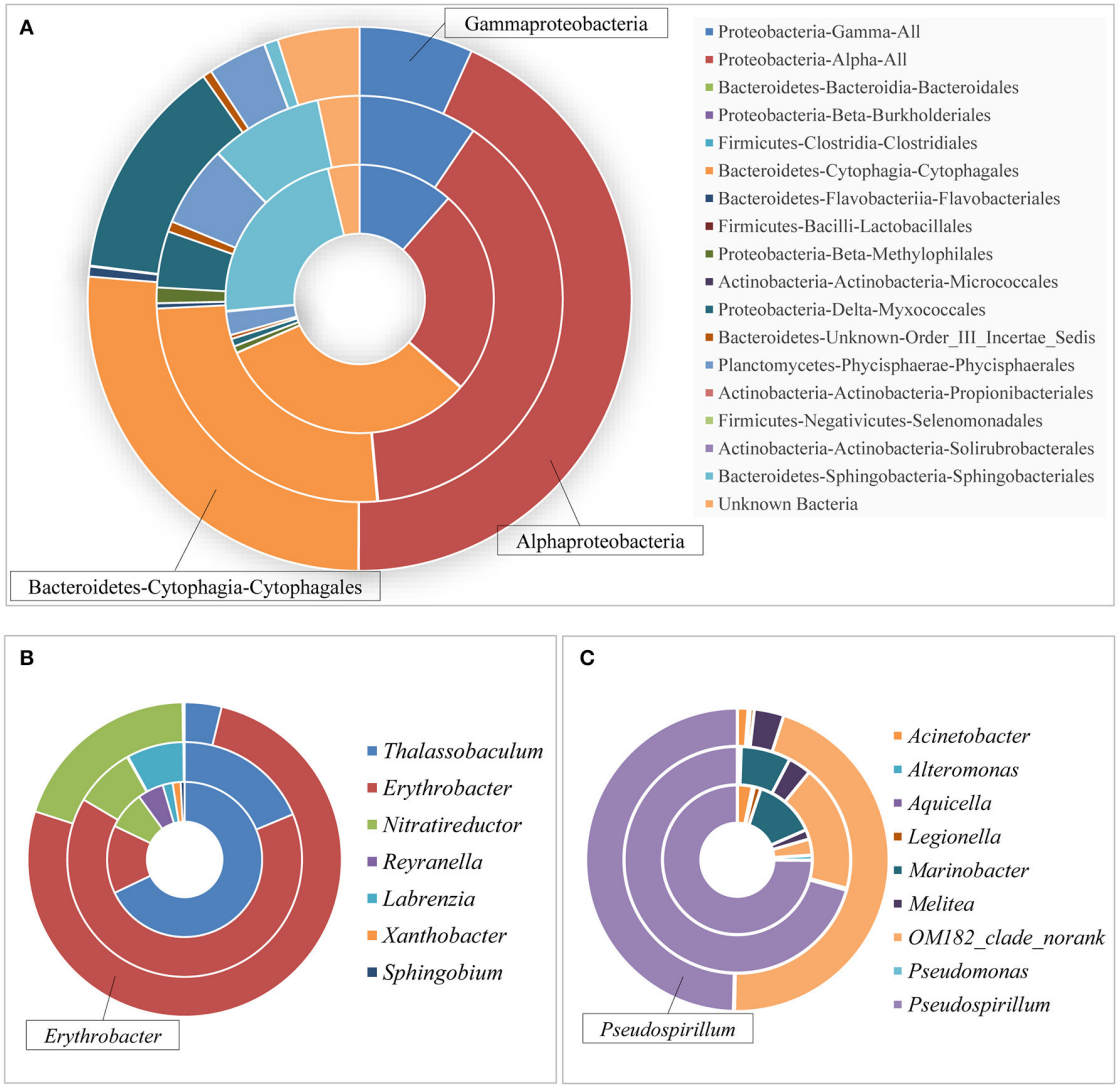
Bacterial community structure in *P. donghaiense* cultures at different taxonomic levels. **(A)** Presentation of group C (the inner loop), LG (the intermediate loop), and HG (the outer loop) in the Phylum-Class-Order level; **(B)** Alphaproteobacteria; and **(C)** Gammaproteobacteria.

Taxonomic analysis showed a shift of bacterial communities at the order and even genus level (Figure 6). Apparently, Cytophagales (31.92%, Figure 6A, the inner loop) dominated the control group, while Alphaproteobacteria constituted the most part of both LG (39.07%, Figure 6A, the intermediate loop) and HG (43.28%, Figure 6A, the outer loop). As one of the major contributors in all microbial groups, only *Rapidithrix* spp. were identified in the order of Cytophagales and its abundance did not change much in the cultures amended with glyphosate compared to the control; thus, we analyzed the community structure of Alphaproteobacteria and Gammaproteobacteria. For Alphaproteobacteria, of those whose abundances increased as glyphosate concentrations increased, the dominant species shifted from *Thalassobaculum* spp. in the control (Figure 6B, the inner loop) to *Erythrobacter* spp. in LG and HG (Figure 6B, the intermediate and outer loops). Additionally, glyphosate even caused some species (*Sphingobium* spp. and *Labrenzia* spp.) to disappear at the end. This illustrated that only a few species were responsible for the increasing of Alphaproteobacteria stimulated by glyphosate, while most of other species were inhibited significantly. As for Gammaproteobacteria, *Pseudospirillum* spp. lost its predominant status gradually as the concentration of glyphosate increased (Figure 6C) and most species' abundances decreased significantly (such as *Marinobacter* spp.). This class of bacteria showed the opposite pattern compared to Alphaproteobacteria: glyphosate boosted the abundance of several lineages (such as *Acinetobacter* spp.) but decreased that of most lineages ultimately causing both its diversity and richness to drop.

## Discussion

Although glyphosate has been used widely, we still know little about its potential ecological impacts on microbial communities associated with phytoplankton. In this study we explored how the phosphorus-containing herbicide influences the bacterial community associated with the HAB-forming dinoflagellate species *P. donghaiense*.

### Bacterial community changes induced by glyphosate

Results from Sanger sequencing of the cloned libraries indicated a significant shift in the bacteria community present in the *P. donghaiense* cultures. In the glyphosate-treated cultures, Alphaproteobacteria and Planctomycetes were promoted markedly, because the abundance of the former increased significantly in LG and HG compared with the control and Planctomycetes were only detected in HG. In contrast, Gammaproteobacteria showed reduced abundance with the presence of glyphosate. These results indicate that Alphaproteobacteria and Planctomycetes responded positively while Gammaproteobacteria responded negatively to glyphosate. The latter is at odds with previous studies that showed Gammaproteobacteria were able to utilize glyphosate as sole P-source (Moore et al., 1983; Quinn et al., 1988). We further sequenced the V4-V5 region in 16S rRNA gene using Illumina high-throughput sequencing for a more complete picture of the microbial community in the *P. donghaiense* cultures.

The high-throughput sequencing result showed that the most abundant phylotypes shared among these three groups belonged to the family Rhodobacteraceae (Figure 3), which are aquatic bacteria that frequently thrive in the marine environments (Pujalte et al., 2014). It is reported that Rhodobacteraceae is one of the three most dominant algal bloom-associated bacterial classes and several laboratory studies have identified specific associations between phytoplankton and certain species of Rhodobacteraceae, making it a major model for the study of microorganism-phytoplankton interactions (review in Buchan et al., 2014). The presence of this group of bacteria in all treatment groups suggests that Rhodobacteraceae is also the major bacteria co-occurring with *P. donghaiense*.

Among the five clusters we detected, clusters C and E responded to glyphosate positively (Figure 4) indicating that they are better adapted to glyphosate and may be able to metabolize glyphosate. Most of the bacteria in these two clusters came from Alphaproteobacteria and Gammaproteobacteria. Other phylotypes identified in these two clusters included Firmicutes, Flavobacteria, Actinobacteria, and Planctomycetes. Of these, Flavobacteria contains species that are usually most abundant in coastal waters and in phytoplankton blooms (reviews in Buchan et al., 2014), suggesting these marine bacteria, if proven to be able to utilize glyphosate, are likely to play an important role in glyphosate metabolism.

By contrast, cluster A showed hormetic response to glyphosate, a dose response phenomenon characterized by a low dose stimulation and high dose inhibition (Axelrod et al., 2004). For instance, *Methylotenera* spp. in cluster A was more abundant in LG than in the control but its abundance decreased by 70-fold in HG, suggesting that *Methylotenera* spp. is resistant to glyphosate at micromolar concentrations but sensitive at millimolar levels. This is common in bacteria: the growth of many cyanobacteria strains were only inhibited by glyphosate at concentrations exceeding 0.3 mM (Forlani et al., 2008). *Pseudomonas* and many other bacteria isolated from sprayed sites all showed high glyphosate resistance even under the concentration of 10 mM (Quinn et al., 1988; Staub et al., 2012). Furthermore, cluster B showed another dose-dependent response to glyphosate. Almost all of these phylotypes were from the aquatic environment and are usually abundant in coastal regions and phytoplankton blooms (Bratbak et al., 1996; Fandino et al., 2001; Pinhassi et al., 2004; Alonso et al., 2007; Buchan et al., 2014). They all showed dose-dependent increases in mortality caused by glyphosate and this dose-dependent induction effect is commonly found in some cyanobacteria and eukaryotic algae (Forlani et al., 2008; Lipok et al., 2010) as well as fish and other amphibious (Annett et al., 2014). Additionally, glyphosate could cause reduction (or disappearance) of some lineages of bacteria such as *Pseudomonas* spp., *Legionella* spp., *Aquarelle* spp., *Xanthobacter* spp., and *Marinobacter* spp., suggesting that they are quite sensitive to glyphosate. Majority of them belong to Gammaproteobacteria, which has been described above as being able to degrade glyphosate. This indicates that glyphosate could change the bacterial composition significantly. Moreover, shifts in microbial community composition were also observed in soil samples exposed to high concentrations of glyphosate (>10 mM; Araújo et al., 2003; Ratcliff et al., 2006; Lancaster et al., 2010). Our study shows that glyphosate can also exact influence on the structure of marine bacteria associated with phytoplankton. All the results in concert support our first hypothesis that glyphosate can induce a shift in bacterial community associated with *P. donghaiense*.

### Glyphosate induced bacteria and *phnJ* gene to promote *P. donghaiense* culture bloom

If an alga could utilize glyphosate, an immediate growth effect would be observed on the alga, as previously reported (Wang et al., 2016). Therefore, our observation that *P. donghaiense* did not grow in the first 60 days clearly indicates that this dinoflagellate is unable to utilize glyphosate directly. This result is consistent with the previous finding that most eukaryotic phytoplankton examined especially dinoflagellates could not utilize glyphosate or other phosphonates (Cui et al., 2015; Wang et al., 2016). So far many cyanobacteria and heterotrophic bacteria have been shown to be able to hydrolyze glyphosate to scavenge phosphate or use the organic carbon (Hove-Jensen et al., 2014), while only few eukaryotic species have been shown to be able to grow with glyphosate provided as sole P-source (Wang et al., 2016). Our observation that *P. donghaiense* showed remarkable growth after 120 days suggests that after the DIP was used up, some specific lineages of bacteria were induced by glyphosate and grew and degraded glyphosate to release sufficient phosphate to support the growth of *P. donghaiense*. This suggests that the bacteria might have originally been in the culture of *P. donghaiense* in very low abundance. Although it is also possible that these bacteria might have been introduced from outside the culture subsequently, this is not so likely because the cultures have not been disturbed since day 10.

Both our physiological and molecular data support our second hypothesis that the bacterial community shift induced by glyphosate involves the increase in glyphosate-degrading species. First, the most remarkably induced groups of bacteria (Clusters A and C) in the presence of glyphosate were from Alphaproteobacteria and Gammaproteobacteria, which have already been reported to utilize glyphosate as a sole P-source (Sviridov et al., 2014). Other phylotypes identified in these two clusters included Firmicutes, Flavobacteria, Actinobacteria, and Planctomycetes. Several species in Firmicutes have been shown to contain the *phn* cluster, a group of genes responsible for phosphonate degradation (Huang et al., 2005). Furthermore, the phosphonatase pathway genes responsible for phosphonate degradation were also detected in many species in Alphaproteobacteria, Gammaproteobacteria, Actinobacteria, and Planctomycetes. Planctomycetes have also been shown to utilize 2-AEP and methylphosphonate as a sole P-source to support growth (Martinez et al., 2010).

Second, a search for *phn* genes (the C-P lyase pathway) against available DNA and protein sequence databases also found significant hits to *phnJ* (the core gene in C-P lyase pathway) in Alphaproteobacteria (Villarreal-Chiu et al., 2012). Furthermore, our qPCR results indicated increase in the *phnJ* gene copy number in the glyphosate treated groups. The *phnJ* copy number from the samples might have been underestimated, however, because the degenerate primers we used were designed from only sequences of Gammaproteobacteria (Table S2), leading to only a dominant *phnJ* sequence for qPCR primer design. However, the Gammaproteobacteria were a dominant group of the bacteria in our cultures and the increasing trend of *phnJ* copy number in the glyphosate-treated cultures was likely representative of the general trend of *phnJ* gene abundance under glyphosate treatment. In addition, our Tax4Fun analysis predicted that the abundance of genes in the bacterial assemblage involved in phosphonates uptake and metabolism consistently increased as glyphosate concentration went up, with only one exception (Table S1). This too might have underestimated the ability of the bacterial assemblage to degrade glyphosate because Tax4Fun can only cover taxa that are represented in the genome database and yet not all of our retrieved 16S rRNA sequence data could be mapped to the existing genome database. This is evident as *phnJ*, which was detected in our cloning and qPCR analyses, was not in the predicted genes (Table S1). Nevertheless, our observation of significantly higher abundances of the predicted glyphosate-metabolizing genes (except one) in glyphosate-amended cultures when compared to the control is indicative of an increase in glyphosate-degrading bacteria; thus, supporting our second hypothesis. In sum, presence of glyphosate in the environment can impact the acquisition of P by the co-occurring phytoplankton species on the one hand and affect the microbial processes in the marine ecosystem on the other.

## Conclusion

Glyphosate represents the simplest chemically synthesized phosphonates that may be flushed into coastal regions. Its discharge and accumulation in the coastal ecosystem could lead to changes in the microbial community structure and impact P nutrient availability for algal proliferation and even bloom formation. For *P. donghaiense*, during the late (stationary) phase of our cultures when phosphate was depleted, glyphosate could serve as a P-source by the aid of the co-occurring bacteria. However, due to the difficulty to determine the composition, residence time, and concentration of DOP, especially glyphosate and other phosphonates that are discharged into the coastal waters, the magnitude of the potential ecological effects of glyphosate and other phosphonates on coastal marine ecosystems still remains to be quantified.

## Author contributions

CW, XL, LL, and SL: Designed the research; CW: Performed the laboratory work and data analysis; LXL: Helped with the data analysis; CW and SL: Wrote the paper.

### Conflict of interest statement

The authors declare that the research was conducted in the absence of any commercial or financial relationships that could be construed as a potential conflict of interest.

## Supplementary material

Table S1 Genes involved in phosphonate metabolism predicted from our 16S rDNA dataset with the Tax4Fun tool and the relative abundance in each treatment.

Table S2 Sequences used in this study for *phnJ* primer design.

Figure S1 Alignment of Gammaproteobacterial sequences from Table S2 and the regions used to design the degenerate primers for *phnJ* gene.

Figure S2 Rarefaction curves for total bacterial communities in the three sampling groups, C, LG, and HG at 3% difference threshold.

Figure S3 Cloning sequences from the degenerate primers for *phnJ* gene.

Figure S4 Melting curve and efficiency curve from the qPCR for *phnJ* gene.

## References

[B1] Ali A. Fletcher R. (1978). Phytotoxic action of glyphosate and amitrole on corn seedlings. Can. J. Bot. 56, 2196–2202. 10.1139/b78-263

[B2] Alonso C. Warnecke F. Amann R. Pernthaler J. (2007). High local and global diversity of Flavobacteria in marine plankton. Environ. Microbiol. 9, 1253–1266. 10.1111/j.1462-2920.2007.01244.x 17472638

[B3] Anderson J. M. Macfadyen A. (1975). Role of terrestrial and aquatic organisms in decomposition processes, in Symposium of the British Ecological Society (Leeds: Blackwell Scientific), 474.

[B4] Annett R. Habibi H. R. Hontela A. (2014). Impact of glyphosate and glyphosate-based herbicides on the freshwater environment. J. Appl. Toxicol. 34, 458–479. 10.1002/jat.2997 24615870

[B5] Araújo A. D. Monteiro R. Abarkeli R. (2003). Effect of glyphosate on the microbial activity of two Brazilian soils. Chemosphere 52, 799–804. 10.1016/S0045-6535(03)00266-2 12757780

[B6] Aßhauer K. P. Wemheuer B. Daniel R. Meinicke P. (2015). Tax4Fun: predicting functional profiles from metagenomic 16S rRNA data. Bioinformatics 31, 2882–2884. 10.1093/bioinformatics/btv287 25957349 PMC4547618

[B7] Axelrod D. Burns K. Davis D. Von Larebeke N. (2004). Hormesis”—an inappropriate extrapolation from the specific to the universal. Int. J. Occup. Environ. Health 10, 335–339. 10.1179/oeh.2004.10.3.335 15473091

[B8] Axler R. P. Redfield G. W. Goldman C. R. (1981). The importance of regenerated nitrogen to phytoplankton productivity to phytoplankton productivity in a Subalpine Lake. Ecology 62, 345–354. 10.2307/1936709

[B9] Battaglin W. Meyer M. Kuivila K. Dietze J. (2014). Glyphosate and its degradation product AMPA occur frequently and widely in US soils, surface water, groundwater, and precipitation. J. Am. Water Resour. Assoc. 50, 275–290. 10.1111/jawr.12159

[B10] Battaglin W. A. Rice K. C. Focazio M. J. Salmons S. Barry R. X. (2009). The occurrence of glyphosate, atrazine, and other pesticides in vernal pools and adjacent streams in Washington, DC, Maryland, Iowa, and Wyoming, 2005–2006. Environ. Monit. Assess. 155, 281–307. 10.1007/s10661-008-0435-y 18677547

[B11] Bloesch J. Stadelmann P. Bührer H. (1977). Primary production, mineralization, and sedimentation in the euphotic zone of two Swiss lakes. Limnol. Oceanogr. 22, 511–526. 10.4319/lo.1977.22.3.0511

[B12] Bolger A. M. Lohse M. Usadel B. (2014). Trimmomatic: a flexible trimmer for Illumina sequence data. Bioinformatics 30, 2114–2120. 10.1093/bioinformatics/btu170 24695404 PMC4103590

[B13] Botta F. Lavison G. Couturier G. Alliot F. Moreau-Guigon E. Fauchon N.. (2009). Transfer of glyphosate and its degradate AMPA to surface waters through urban sewerage systems. Chemosphere 77, 133–139. 10.1016/j.chemosphere.2009.05.008 19482331

[B14] Bratbak G. Wilson W. Heldal M. (1996). Viral control of *Emiliania huxleyi* blooms? J. Mar. Syst. 9, 75–81. 10.1016/0924-7963(96)00018-8

[B15] Buchan A. Lecleir G. Gulvik C. Gonzalez J. (2014). Master recyclers: features and functions of bacteria associated with phytoplankton blooms. Nat. Rev. Microbiol. 12:686. 10.1038/nrmicro3326 25134618

[B16] Bunt J. S. (1961). Blue-green algae: growth. Nature 192, 1274–1275. 10.1038/1921274a0 13874676

[B17] Caldwell D. E. Overbeck J. (1977). The planktonic microflora of lakes. Crit. Rev. Microbiol. 5, 305–370. 10.3109/10408417709102809 407052

[B18] Croft M. T. Lawrence A. D. Raux-Deery E. Warren M. J. Smith A. G. (2005). Algae acquire vitamin B12 through a symbiotic relationship with bacteria. Nature 438, 90–93. 10.1038/nature04056 16267554

[B19] Cui Y. Lin X. Zhang H. Lin L. Lin S. (2015). PhnW-PhnX pathway in dinoflagellates not functional to utilize extracellular phosphonates. Front. Mar. Sci. 2:120 10.3389/fmars.2015.00120

[B20] Delucca R. McCracken M. D. (1977). Observations on interactions between naturally-collected bacteria and several species of algae. Hydrobiologia 55, 71–75. 10.1007/BF00034807

[B21] Duke S. O. (2003). Ecophysiological aspects of allelopathy. Planta 217, 529–539. 10.1007/s00425-003-1054-z 12811559

[B22] Duke S. O. Powles S. B. (2008). Glyphosate: a once-in-a-century herbicide. Pest Manag. Sci. 64, 319–325. 10.1002/ps.1518 18273882

[B23] Edgar R. C. (2013). UPARSE: highly accurate OTU sequences from microbial amplicon reads. Nat. Methods 10, 996–998. 10.1038/nmeth.2604 23955772

[B24] Edgar R. C. Haas B. J. Clemente J. C. Quince C. Knight R. (2011). UCHIME improves sensitivity and speed of chimera detection. Bioinformatics 27, 2194–2200. 10.1093/bioinformatics/btr381 21700674 PMC3150044

[B25] Fandino L. Riemann L. Steward G. Long R. Azam F. (2001). Variations in bacterial community structure during a dinoflagellate bloom analyzed by DGGE and 16S rDNA sequencing. Aquat. Microb. Ecol. 23, 119–130. 10.3354/ame023119

[B26] Forlani G. Pavan M. Gramek M. Kafarski P. Lipok J. (2008). Biochemical bases for a widespread tolerance of cyanobacteria to the phosphonate herbicide glyphosate. Plant Cell Physiol. 49, 443–456. 10.1093/pcp/pcn021 18263622

[B27] Giesy J. P. Dobson S. Solomon K. R. (2000). Ecotoxicological risk assessment for Roundup® herbicide, in Reviews of Environmental Contamination and Toxicology, Vol. 167, ed Ware G. M. (New York, NY: Springer).

[B28] Golterman H. (1972). Role of phytoplankton in detritus formation. Mem. Ist. Ital. Idrobiol. 29(Suppl.), 89–103.

[B29] Grube A. Donaldson D. Kiely T. Wu L. (2011). Pesticides Industry Sales and Usage. Washington, DC: US EPA.

[B30] Guillard R. R. (1975). Culture of phytoplankton for feeding marine invertebrates, in Culture of Marine Invertebrate Animals, eds Smith W. L. Chanley M. H. (Greenport, NY: Springer), 29–60.

[B31] Haines K. C. Guillard R. R. (1974). Growth of vitamin B12-requiring marine diatoms in mixed laboratory cultures with vitamin B12-producing marine bacteria12. J. Phycol. 10, 245–252. 10.1111/j.0022-3646.1974.00245.x

[B32] Hall B. G. (2013). Building phylogenetic trees from molecular data with MEGA. Mol. Biol. Evol. 30, 1229–1235. 10.1093/molbev/mst012 23486614

[B33] Hernando F. Royuela M. Mu-oz-Rueda A. Gonzalez-Murua C. (1989). Effect of glyphosate on the greening process and photosynthetic metabolism in Chlorella pyrenoidosa. J. Plant Physiol. 134, 26–31. 10.1016/S0176-1617(89)80197-X

[B34] Hobbie J. Rublee P. (1977). Radioisotope studies of heterotrophic bacteria in aquatic ecosystems, in Aquatic Microbial Communities, ed Cairns J. (New York, NY: Garland), 441–476.

[B35] Hove-Jensen B. Zechel D. L. Jochimsen B. (2014). Utilization of glyphosate as phosphate source: biochemistry and genetics of bacterial carbon-phosphorus lyase. Microbiol. Mol. Biol. Rev. 78, 176–197. 10.1128/MMBR.00040-13 24600043 PMC3957732

[B36] Huang J. Su Z. Xu Y. (2005). The evolution of microbial phosphonate degradative pathways. J. Mol. Evol. 61, 682–690. 10.1007/s00239-004-0349-4 16245012

[B37] Huber T. Faulkner G. Hugenholtz P. (2004). Bellerophon: a program to detect chimeric sequences in multiple sequence alignments. Bioinformatics 20, 2317–2319. 10.1093/bioinformatics/bth226 15073015

[B38] Lancaster S. H. Hollister E. B. Senseman S. A. Gentry T. J. (2010). Effects of repeated glyphosate applications on soil microbial community composition and the mineralization of glyphosate. Pest Manag. Sci. 66, 59–64. 10.1002/ps.1831 19697445

[B39] LeGresley M. McDermott G. (2010). Counting Chamber Methods for Quantitative Phytoplankton Analysis—Haemocytometer, Palmer-Maloney Cell and Sedgewick-Rafter Cell. Microscopic and molecular methods for quantitative phytoplankton analysis. UNESCO (IOC Manuals and Guides), 25–30.

[B40] Lipok J. Studnik H. Gruyaert S. (2010). The toxicity of Roundup® 360 SL formulation and its main constituents: glyphosate and isopropylamine towards non-target water photoautotrophs. Ecotoxicol. Environ. Saf. 73, 1681–1688. 10.1016/j.ecoenv.2010.08.017 20813408

[B41] Magoč T. Salzberg S. L. (2011). FLASH: fast length adjustment of short reads to improve genome assemblies. Bioinformatics 27, 2957–2963. 10.1093/bioinformatics/btr507 21903629 PMC3198573

[B42] Martinez A. Tyson G. W. DeLong E. F. (2010). Widespread known and novel phosphonate utilization pathways in marine bacteria revealed by functional screening and metagenomic analyses. Environ. Microbiol. 12, 222–238. 10.1111/j.1462-2920.2009.02062.x 19788654

[B43] Menzel D. W. Spaeth J. P. (1962). Occurrence of vitamin B12 in the Sargasso Sea. Limnol. Oceanogr. 7, 151–154. 10.4319/lo.1962.7.2.0151

[B44] Moore J. K. Braymer H. D. Larson A. D. (1983). Isolation of a *Pseudomonas* sp. which utilizes the phosphonate herbicide glyphosate. Appl. Environ. Microbiol. 46, 316–320. 16346357 10.1128/aem.46.2.316-320.1983 PMC239379

[B45] Nadin P. (2007). The Use of Plant Protection Products in the European Union. Luxembourg: European Commission. Eurostat. Statistical Office of the European Communities, 35–36.

[B46] Overbeck J. Babenzien H. D. (1964). Bakterien und Phytoplankton eines Kleingewässers im Jahreszyklus. Z. Mikrobiol. 4, 59–76. 10.1002/jobm.3630040106 14298266

[B47] Parsons T. R. (2013). A Manual of Chemical & Biological Methods for Seawater Analysis. New York, NY: Elsevier.

[B48] Pérez G. Torremorell A. Mugni H. Rodriguez P. Vera M. S. do Nascimento M.. (2007). Effects of the herbicide Roundup on freshwater microbial communities: a mesocosm study. Ecol. Appl. 17, 2310–2322. 10.1890/07-0499.1 18213971

[B49] Pesce S. Batisson I. Bardot C. Fajon C. Portelli C. Montuelle B.. (2009). Response of spring and summer riverine microbial communities following glyphosate exposure. Ecotoxicol. Environ. Saf. 72, 1905–1912. 10.1016/j.ecoenv.2009.07.004 19646758

[B50] Pinhassi J. Sala M. Havskum H. Peters F. Guadayol O. Malits A.. (2004). Changes in bacterioplankton composition under different phytoplankton regimens. Appl. Environ. Microbiol. 70, 6753–6766. 10.1128/AEM.70.11.6753-6766.2004 15528542 PMC525254

[B51] Pujalte M. J. Lucena T. Ruvira M. A. Arahal D. R. Macián M. C. (2014). The family Rhodobacteraceae, in The Prokaryotes: Alphaproteobacteria and Betaproteobacteria, eds Rosenberg E. DeLong E. F. Lory S. Stackebrandt E. Thompson F. L. (Heidelberg: Springer Verlag), 439–512.

[B52] Quast C. Pruesse E. Yilmaz P. Gerken J. Schweer T. Yarza P.. (2012). The SILVA ribosomal RNA gene database project: improved data processing and web-based tools. Nucleic Acids Res. 41, D590–D596. 10.1093/nar/gks1219 23193283 PMC3531112

[B53] Quinn J. P. Peden J. M. Dick R. E. (1988). Glyphosate tolerance and utilization by the microflora of soils treated with the herbicide. Appl. Microbiol. Biotechnol. 29, 511–516. 10.1007/BF00269078

[B54] Ratcliff A. W. Busse M. D. Shestak C. J. (2006). Changes in microbial community structure following herbicide (glyphosate) additions to forest soils. Appl. Soil Ecol. 34, 114–124. 10.1016/j.apsoil.2006.03.002

[B55] Rohr J. R. Crumrine P. W. (2005). Effects of an herbicide and an insecticide on pond community structure and processes. Ecol. Appl. 15, 1135–1147. 10.1890/03-5353

[B56] Schaffer J. Sebetich M. (2004). Effects of aquatic herbicides on primary productivity of phytoplankton in the laboratory. Bull. Environ. Contam. Toxicol. 72, 1032–1037. 10.1007/s00128-004-0347-7 15266702

[B57] Schopf J. W. (1975). Precambrian paleobiology: problems and perspectives. Annu. Rev. Earth Planet. Sci. 3:213 10.1146/annurev.ea.03.050175.001241

[B58] Spellerberg I. F. (2008). Shannon-Wiener Index, in Encyclopedia of Ecology, ed Jorgensen S. E. Fath B. (Elsevier B.V.), 3249–3252.

[B59] Staub J. M. Brand L. Tran M. Kong Y. Rogers S. G. (2012). Bacterial glyphosate resistance conferred by overexpression of an *E. coli* membrane efflux transporter. J. Indus. Microbiol. Biotechnol. 39, 641–647. 10.1007/s10295-011-1057-x 22089966

[B60] Sviridov A. Shushkova T. Ermakova I. Ivanova E. Leontievsky A. (2014). Glyphosate: safety risks, biodegradation, and bioremediation, in Current Environmental Issues and Challenges, eds Cao G. Orrù R. (Dordrecht: Springer), 183–195.

[B61] Swift D. G. Guilford R. R. (1978). Unexpected response to vitamin B12 of dominant centric diatoms from the spring bloom in the Gulf Of Maine (Northeast Atlantic Ocean). J. Phycol. 14, 377–386. 10.1111/j.1529-8817.1978.tb02456.x

[B62] Villarreal-Chiu J. F. Quinn J. P. McGrath J. W. (2012). The genes and enzymes of phosphonate metabolism by bacteria, and their distribution in the marine environment. Front. Microbiol. 3:19. 10.3389/fmicb.2012.00019 22303297 PMC3266647

[B63] Wang C. Lin X. Li L. Lin S. (2016). Differential growth responses of marine phytoplankton to herbicide glyphosate. PLoS ONE 11:e0151633. 10.1371/journal.pone.0151633 26985828 PMC4795549

[B64] Wang Q. Garrity G. M. Tiedje J. M. Cole J. R. (2007). Naive Bayesian classifier for rapid assignment of rRNA sequences into the new bacterial taxonomy. Appl. Environ. Microbiol. 73, 5261–5267. 10.1128/AEM.00062-07 17586664 PMC1950982

[B65] Webster G. Newberry C. J. Fry J. C. Weightman A. J. (2003). Assessment of bacterial community structure in the deep sub-seafloor biosphere by 16S rDNA-based techniques: a cautionary tale. J. Microbiol. Methods 55, 155–164. 10.1016/S0167-7012(03)00140-4 14500007

[B66] Winer B. J. Brown D. R. Michels K. M. (1971). Statistical Principles in Experimental Design. New York, NY: McGraw-Hill.

[B67] Xu J. Song X.-C. Zhang Q. Pan H. Liang Y. Fan X.-W.. (2011). Characterization of metal removal of immobilized Bacillus strain CR-7 biomass from aqueous solutions. J. Hazard. Mater. 187, 450–458. 10.1016/j.jhazmat.2011.01.047 21300432

[B68] Zhang H. Bhattacharya D. Lin S. (2005). Phylogeny of dinoflagellates based on mitochondrial cytochrome B and nuclear small subunit rDNA sequence comparisons. J. Phycol. 41, 411–420. 10.1111/j.1529-8817.2005.04168.x

[B69] Zhou G. Shi Q. S. Ouyang Y. S. Chen Y. B. (2014). Involvement of outer membrane proteins and peroxide-sensor genes in Burkholderia cepacia resistance to isothiazolone. World J. Microbiol. Biotechnol. 30, 1251–1260. 10.1007/s11274-013-1538-3 24197783

