# Supplementary material for: Glyphosate Shapes a Dinoflagellate-Associated Bacterial Community While Supporting Algal Growth as Sole Phosphorus Source
Source: Front Microbiol. 2017 Dec 19;8:2530. doi: 10.3389/fmicb.2017.02530 (PMC5742145; doi:10.3389/fmicb.2017.02530)
Supplement: Figure S1 — Alignment of Gammaproteobacterial sequences from Table S2 and the regions used to design the degenerate primers for phnJ gene. [file Image1.PDF]

```
Alignment: /Users/Lin/Desktop/phnJ.fas
Seaview [blocks=8 fontsize=8 A4-landscape] on Tue Oct 17 10:46:32 2017

                  1
EscCo_NC_000913.3 ATGGCTAA ---TC--- -------- -------- -------- -------- -TGAGC-- -------- -------- -------- ------GG CTACAACT TTGCCTAC CTCGACGA GCAGACCA AACGCATG
YerPe_NC_003143.1 ATGACTGA GGTTC--- -------- -------- -------- -------- -TCACT-- -------- -------- -------- ------GG CTACAACC TGGGTTAT TTGGATGA GCAAACCA AGTGTACA
YerEn_NC_008800.1 ATGACTGA AGTTC--- -------- -------- -------- -------- -TCACC-- -------- -------- -------- ------GG TTACAATC TGGGTTAT CTGGACGA GCAAACCA AAAGAACA
ShiDy_NC_007606.1 ATGGCTAA ---TC--- -------- -------- -------- -------- -TGAGC-- -------- -------- -------- ------GG CTACAACT TTGCGTAC CTCGACGA GCAGACCA AACGCATG
PseAe_NC_022360.1 ATGAGCCT --TTC--- -------- -------- -------- -----CCC GCAAGCCA GCGCCG-- -------- ---CGCCG GAGCAGGG CTACAACT TCGCCTAC CTCGACGA GCAGACCA AGCGCATG
PseAe_NC_022361.1 ATGAGCCT --TTC--- -------- -------- -------- -----CCC GCAAGCCA GCGCCG-- -------- ---CGCCG GAGCAGGG CTACAACT TCGCCTAC CTCGACGA GCAGACCA AGCGCATG
EntCl_NC_014121.1 ATGGCTAA ---CC--- -------- -------- -------- -------- -TAAGC-- -------- -------- -------- ------GG CTACAACT TTGCTTAT CTGGACGA GCAAACCA AACGCATG
PseSy_NC_004578.1 ATGAACAC ---TC--- -------- -------- -------- -----CAG TCAAGAGC GCTGCACA GACCCGAC CAGCGCGA GATGAGGC GTACAACT TCGCGTAT CTGGACGA ACAGACCA AACGCATG
PseAe_NC_022594.1 ATGAGCCT --TTC--- -------- -------- -------- -----CCC GCAAGCCA GCGCCG-- -------- ---CGCCG GAGCAGGG CTACAACT TCGCCTAC CTCGACGA GCAGACCA AGCGCATG
PseAe_NC_022591.1 ATGAGCCT --TTC--- -------- -------- -------- -----CCC GCAAGCCA GCGCCG-- -------- ---CGCCG GAGCAGGG CTACAACT TCGCCTAC CTCGACGA GCAGACCA AGCGCATG
VibNi_NC_022528.1 ATGAGTAA --TTCGAC GCAACAAA GCCATGCA TCAATACA TGACGCAC CTGAACTT GCTCTGAC GAGCAATA CTCAGGCA ATACAGGG CTACAACT ATGGCTAC CTCGATGA GCAGACAA AACGCATG
EscCo_NC_011751.1 ATGGCTAA ---TC--- -------- -------- -------- -------- -TGAGC-- -------- -------- -------- ------GG CTACAACT TTGCCTAC CTCGACGA GCAGACCA AACGCATG
EscCo_NC_011750.1 ATGGCTAA ---TC--- -------- -------- -------- -------- -TGAGC-- -------- -------- -------- ------GG CTACAACT TTGCCTAC CTCGACGA GCAGACCA AACGCATG

                                                                                                  PhnJF1
                  129
EscCo_NC_000913.3 ATCCGCCG CGCCATCT TAAAAGCG GTGGCGAT CCCCGGTT ATCAGGTG CCGTTTGG CGGGCGCG AGATGCCG ATGCCGTA CGGCTGGG GAACCGGC GGCATTCA GCTTACCG CCAGCGTG ATTGGCGA
YerPe_NC_003143.1 TTGCGCCG TGCCATAC TGAAAGCC GTCGCAAT CCCAGGCT ATCAAGTG CCATTTGG TGGGCGTG AGATGCCA ATGCCTTA CGGCTGGG GGACTGGC GGCATTCA GTTGACGG CGTGTCTG ATTGGCCA
YerEn_NC_008800.1 TTGCGCCG TGCGTTAC TGAAAGCC GTGGCCAT CCCCGGTT ATCAGGTG CCATTCGG CGGGCGTG AGATGCCG ATGCCTTA TGGTTGGG GCACCGGT GGCATTCA GTTAACCG CCTGCCTG ATTGGCCG
ShiDy_NC_007606.1 AGCCGCCG CGCCATCT TAAAAGCG GTAGCGAT CCCCGGTT ATCAGGTA CCGTTTGG CGGGCGCG AGATGCCA ATGCCGTA CGGCTGGG GAACCGGC GGCATACA GCTCACCG CCAGCGTG ATTGGCGA
PseAe_NC_022360.1 ATCCGCCG CGGCCTGC TCAAGGCG GTGGCGAT CCCCGGCT ACCAGGTG CCCTTCGG CGGCCGCG AGATGCCG CTGCCCTA CGGCTGGG GCACCGGC GGCATGCA GGTGACCG CGGCCATC CTCGGTGC
PseAe_NC_022361.1 ATCCGCCG CGGCCTGC TCAAGGCG GTGGCGAT CCCCGGCT ACCAGGTG CCCTTCGG CGGCCGCG AGATGCCG CTGCCCTA CGGCTGGG GCACCGGC GGCATGCA GGTGACCG CGGCCATC CTCGGTGC
EntCl_NC_014121.1 ATCCGCCG CGCCATCC TGAAAGCG GTCGCTAT CCCCGGCT ATCAGGTG CCGTTTGG TGGTCGAG AAATGCCG ATGCCTTA CGGCTGGG GCACCGGG GGCATTCA GCTTACCG CCAGCGTC ATCGGTGA
PseSy_NC_004578.1 ATTCGCCG TGCCTTGC TCAAGGCC GTCGCGAT TCCCGGTT ATCAGGTG CCATTCGG CGGGCGCG AAATGCCG TTGCCTTA CGGCTGGG GCACCGGT GGCATGCA GCTGACGG CGGCGATC CTGGGTGA
PseAe_NC_022594.1 ATCCGCCG CGGCCTGC TCAAGGCG GTGGCGAT CCCCGGCT ACCAGGTG CCCTTCGG CGGCCGCG AGATGCCG CTGCCCTA CGGCTGGG GCACCGGC GGCATGCA GGTGACCG CGGCCATC CTCGGTGC
PseAe_NC_022591.1 ATCCGCCG CGGCCTGC TCAAGGCG GTGGCGAT CCCCGGCT ACCAGGTG CCCTTCGG CGGCCGCG AGATGCCG CTGCCCTA CGGCTGGG GCACCGGC GGCATGCA GGTGACCG CGGCCATC CTCGGTGC
VibNi_NC_022528.1 ATTCGTCG CGCCATTT TAAAAGCC GTTGCCAT TCCGGGGT ATCAGGTG CCATTTGG CGGTCGCG AAATGCCC ATGCCTTA TGGTTGGG GCACAGGC GGTATACA GATTACTG CGTCCATT ATCGGCAC
EscCo_NC_011751.1 ATCCGCCG CGCCATCT TAAAAGCG GTAGCGAT CCCCGGTT ATCAGGTA CCTTTTGG CGGGCGCG AGATGCCG ATGCCGTA CGGCTGGG GAACTGGT GGTATCCA GCTTACCG CCAGCGTG ATTGGCGA
EscCo_NC_011750.1 ATCCGCCG CGCCATCT TAAAAGCG GTGGCGAT CCCCGGTT ATCAGGTG CCGTTTGG CGGGCGCG AGATGCCG ATGCCGTA CGGCTGGG GAACTGGT GGTATCCA GCTTACCG CCAGCGTG ATTGGCGA

                  257
EscCo_NC_000913.3 AAGCGACG TGCTGAAG GTGATTGA CCAGGGCG CGGATGAC ACCACCAA CGCCGTGT CGATTCGC AACTTCTT CAAGCGCG TGACCGGG GTAAACAC CACGGAAC GTACGGAC GATGCGAC GGTTATCC
YerPe_NC_003143.1 CACGGACG TGTTGAAA GTCATTGA TCAGGGCG CAGATGAC ACCACCAA TGCCGTCT CGATCCGC CGCTTTTT CCAACGGG TCAGCGGG GTGGCAAC CACAGAAA AAACCACT GAAGCCAC ATTAATCC
YerEn_NC_008800.1 TTACGATG TGCTGAAA GTGATCGA CCAGGGAG CCGATGAC ACCACTAA TGCGGTGT CGATCCGC CGCTTCTT CCAGCGGG TCAGCGGA GTGGCGAC TACCGAAA AAACCACT AACGCCAC CTTGATCC
ShiDy_NC_007606.1 AAGCGACG TGTTGAAG GTGATTGA CCAGGGCG CGGATGAC ACCACCAA CGCCGTGT CGATCCGC AACTTCTT TAAGCGCG TGACCGGG GTGAATAC CACCGAAC GCACGGAT GATGCGAC GGTTATCC
PseAe_NC_022360.1 CGACGACG TGCTCAAG GTGATCGA CCAGGGCG CCGACGAC ACCACCAA CGCGGTAT CCATCCGG CGTTTCTT CGCCCGCA CCGCCGGG GTCGCCAC CACCGAAC GGACCCCG GAGGCGAC GGTGATCC
PseAe_NC_022361.1 CGACGACG TGCTCAAG GTGATCGA TCAGGGCG CCGACGAC ACCACCAA CGCGGTAT CCATCCGG CGTTTCTT CGCCCGCA CCGCCGGG GTCGCCAC CACCGAAC GGACCCCG GAGGCGAC GGTGATCC
EntCl_NC_014121.1 GTCAGACG TGCTGAAG GTCATCGA CCAGGGAG CCGACGAC ACCACCAA CGCCGTGT CGATTCGG AACTTCTT TAAGCGCG TGACGGGC GTCAACAC CACCGAAA AAACCGAA GATGCGAC GCTTATCC
PseSy_NC_004578.1 CGACGACG TGCTCAAA GTCATCGA TCAGGGCG CTGACGAT ACCACCAA CGCGGTGT CGATCCGG CGCTTCTT TGCCCGTA CCGCAGGT ATCGCCAC CACCGAGC GCACCCCG GATGCCAC GGTCATCC
PseAe_NC_022594.1 CGACGACG TGCTCAAG GTGATCGA TCAGGGCG CCGACGAC ACCACCAA CGCGGTAT CCATCCGG CGTTTCTT CGCCCGCA CCGCCGGG GTCGCCAC CACCGAAC GGACCCCG GAGGCGAC GGTGATCC
PseAe_NC_022591.1 CGACGACG TGCTCAAG GTGATCGA TCAGGGCG CCGACGAC ACCACCAA CGCGGTAT CCATCCGG CGTTTCTT CGCCCGCA CCGCCGGG GTCGCCAC CACCGAAC GGACCCCG GAGGCGAC GGTGATCC
VibNi_NC_022528.1 CGACGACA CGCTCAAG GTGATTGA TCAAGGAG CAGACGAC ACCACCAA TGCAGTAT CGATTCGC AAATTCTT TGAAAAAG TCGCTCAC GCCGACAC CACTGAGA AAACCGAT GCCGCCAG CATTATCC
EscCo_NC_011751.1 AAGCGACG TGTTGAAG GTGATTGA CCAGGGTG CGGATGAC ACCACCAA CGCCGTGT CGATTCGC AACTTCTT TAAGCGCG TGACCGGG GTAAACAC CACTGAAC GTACGGAC GATGCGAC GCTTATCC
EscCo_NC_011750.1 AAGCGATG TGCTGAAG GTGATTGA CCAGGGCG CGGACGAC ACCACCAA CGCGGTGT CGATCCGT AACTTCTT TAAGCGCG TGACCGGG GTAAACAC TACCGAAC GCACGGAC GATGCGAC GCTTATCC

                                                                                                                                                PhnJR1
                  385
EscCo_NC_000913.3 AGACGCGT CACCGCAT CCCCGAAA CGCCGCTG ACCGAAGA TCAGATAA TTATCTTC CAGGTGCC AATCCCCG AGCCGCTG CGCTTTAT CGAGCCGC GCGAAACG GAAACCCG CACCATGC ACGCGCTG
YerPe_NC_003143.1 AGACCCGC CATCGCAT ACCGGAAA CCCCACTG ACAGAAGA TCAGATCC TGATTTAT CAGGTACC GATCCCAG AACCGCTG CGATTTAT TGAGCCAC GTGAAACC GAGACTCG AAAAATGC ATGCACTG
YerEn_NC_008800.1 AGACCCGC CACCGTAT TCCAGAAA CGCCGCTG ACGGAGGA TCAGATCC TTATCTAT CAGGTACC GATCCCAG AGCCGCTG CGCTTTAT TGAACCGC GTGAAACC GAGACGCG CAAAATGC ATGCGCTG
ShiDy_NC_007606.1 AGACGCGT CACCGCAT CCCCGAAA CGCCGCTG ACCGAAGA TCAGATAA TTATCTTC CAGGTGCC GATCCCCG AACCGCTG CGCTTTAT CGAGCCGC GCGAAACG GAAACCCG CACCATGC ACGCGCTG
PseAe_NC_022360.1 AGACCCGC CACCGGAT ACCGGAAA CCGCCCTG AGCGGCGA GCAGATCA TGGTCTAC CAGGTGCC GATCCCCG AGCCGCTG CGCTTCAT CGAGCCTT CCGAGGCG GAGACCCG GACCATGC ATGCGCTC
PseAe_NC_022361.1 AGACCCGC CACCGGAT ACCGGAAA CCGCCCTG AGCGGCGA GCAGATCA TGGTCTAC CAGGTGCC GATCCCCG AGCCGCTG CGCTTCAT CGAGCCTT CCGAGGCG GAGACCCG GACCATGC ATGCGCTC
EntCl_NC_014121.1 AGACCCGC CACCGTAT TCCCGAAA CGCCGCTC ACTGAAGA TCAGATTT TGATTTTC CAGGTGCC GATCCCGG AGCCGCTG CGCTTTAT CGAGCCGC GCGAAACA GAAACCCG CACCATGC ACGCCCTG
PseSy_NC_004578.1 AGACCCGC CACCGGAT TCCGGAAA CACCGCTG CACGCTGA TCAGATCA TGGTCTAT CAAGTGCC GATCCCCG AGCCGCTG CGTTTCAT CGAACCCT CGGAAACC GAGACCCG CACGATGC ACGCGCTG
PseAe_NC_022594.1 AGACCCGC CACCGGAT ACCGGAAA CCGCCCTG AGCGGCGA GCAGATCA TGGTCTAC CAGGTGCC GATCCCCG AGCCGCTG CGCTTCAT CGAGCCTT CCGAGGCG GAGACCCG GACCATGC ATGCGCTC
PseAe_NC_022591.1 AGACCCGC CACCGGAT ACCGGAAA CCGCCCTG AGCGGCGA GCAGATCA TGGTCTAC CAGGTGCC GATCCCCG AGCCGCTG CGCTTCAT CGAGCCTT CCGAGGCG GAGACCCG GACCATGC ATGCGCTC
VibNi_NC_022528.1 AAACGCGT CACCGCAT TCCAGAAA AGCCACTC AAAGAAGG ACAAATTC TGGTGTAT CAAGTGCC GATTCCAG AGCCATTG CGCTTCTT AGAGCCGA GAGAAACG GAAACCCG AAAAATGC ACGCGCTA
EscCo_NC_011751.1 AGACGCGT CACCGCAT CCCCGAAA CGCCGCTG ACCGAAGA TCAGATCA TTATCTTC CAGGTGCC AATCCCGG AACCGCTG CGCTTTAT CGAGCCGC GCGAAACG GAAACCCG CACTATGC ACGCGCTG
EscCo_NC_011750.1 AGACGCGC CATCGCAT CCCCGAAA CGCCGCTC ACCGAAGA TCAGATCA TTATTTTC CAGGTGCC GATCCCCG AACCGCTG CGCTTTAT CGAGCCGC GCGAAACG GAAACCCG CACCATGC ACGCGCTG
```

```
                  PhnJR1
                  513
EscCo_NC_000913.3 GAAGAGTA CGGCGTGA TGCAGGTG AAACTGTA TGAAGATA TCGCCCGC TTCGGTCA TATCGCCA CCACCTAC GCCTATCC GGTGAAGG TAAATGGG CGCTACGT GATGGACC CGTCGCCG ATCCCGAA
YerPe_NC_003143.1 GAGGAGTA CGGCGTGA TGCAGGTA AAACTGTA CGAAGATA TCGCCCGC TACGGCCA TATCGCCA CCACCTAT GCCTACCC GGTCAAAG TGAATGAT CGCTATGT CATGGACC CTTCACCG ATCCCGAA
YerEn_NC_008800.1 GAGGAATA CGGCGTAA TGCAAGTC AAGCTATA TGAAGATA TCGCTCGC TACGGCCA TATCGCGA CCACCTAC GCCTATCC GGTAAAAG TGAATGAT CGCTATGT GATGGATC CGTCGCCA ATTCCGAA
ShiDy_NC_007606.1 GAGGAGTA CGGCGTGA TGCAGGTG AAGCTGTA TGAAGATA TCGCCCGC TTCGGTCA TATCGCCA CCACCTAC GCCTATCC AGTGAAGG TAAATGGG CGCTACGT GATGGACC CGTCGCCG ATCCCGAA
PseAe_NC_022360.1 GACGACTA CGGGGTGA TGCACGTG AAGCTCTA CGAGGACA TCGCCACC TTCGGCCA TATCGCCA CCAGCTAT GCCTACCC GGTTACGG TGGACGAG CGCTACGT GATGGATC CCTCGCCA ATTCCCAA
PseAe_NC_022361.1 GACGACTA CGGGGTGA TGCACGTG AAGCTCTA CGAGGACA TCGCCACC TTCGGCCA TATCGCCA CCAGCTAT GCCTACCC GGTGACGG TGGACGAG CGCTACGT GATGGATC CCTCGCCG ATTCCCAA
EntCl_NC_014121.1 GAAGAGTA CGGGGTGA TGCAGGTG AAGCTGTA TGAAGATA TCGCCCGC TTCGGCCA TATCGCCA CCACCTAC GCCTACCC GGTGAAGG TCAACGGG CGCTACGT GATGGATC CGTCGCCG ATCCCGAA
PseSy_NC_004578.1 GATGACTA CGGCGTCA TGCACGTC AAACTCTA CGAAGACA TCGCCACC TTCGGCCA CATCGCTA CCAGCTAT GCCTATCC GGTGATGG TCGATGAG CGTTACGT GATGGACC CCTCGCCG ATTCCGAA
PseAe_NC_022594.1 GACGACTA CGGGGTGA TGCACGTG AAGCTCTA CGAGGACA TCGCCACC TTCGGCCA TATCGCCA CCAGCTAT GCCTACCC GGTGACGG TGGACGAG CGCTACGT GATGGATC CCTCGCCG ATTCCCAA
PseAe_NC_022591.1 GACGACTA CGGGGTGA TGCACGTG AAGCTCTA CGAGGACA TCGCCACC TTCGGCCA TATCGCCA CCAGCTAT GCCTACCC GGTGACGG TGGACGAG CGCTACGT GATGGATC CCTCGCCG ATTCCCAA
VibNi_NC_022528.1 AGTGAATA CGGGATCA TGCACGTG AAGCTTTA TGAAGACA TAGCCCGT TACGGGCA CATCTCGA CCTCTTAC GCCTACCC GGTCAAGG TGAACCAG CGATACAT CATGGACC CGTCTCCG ATTCCAAA
EscCo_NC_011751.1 GAGGAGTA CGGCGTGA TGCAGGTG AAACTGTA TGAAGATA TCGCCCGC TTCGGTCA TATCGCCA CCACCTAT GCCTATCC GGTGAAGG TGAACGGG CGCTACGT GATGGACC CGTCGCCG ATCCCGAA
EscCo_NC_011750.1 GAGGAGTA CGGCGTGA TGCAGGTG AAACTGTA TGAAGATA TCGCCCGC TTCGGTCA TATCGCCA CCACCTAT GCCTATCC GGTGAAGG TGAACGGG CGCTACGT GATGGACC CGTCGCCG ATCCCGAA

                  641
EscCo_NC_000913.3 ATTCGATA ACCCAAAA ATGGACAT GATGCCCG CCCTGCAA CTGTTCGG CGCGGGGC GCGAGAAG CGCATCTA TGCGGTGC CGCCGTTT ACCCGCGT GGAAAGTC TCGATTTC GACGATCA CCCGTTCA
YerPe_NC_003143.1 ATTTGATA ATCCCAAA ATGCATAT GATGCCCG CACTGCAA CTGTTCGG GGCGGGTC GTGAGAAA CGCCTTTA TGCCCTGC CCCCGTTT ACCAAGGT GGAAAGCC TCGATTTT GACGATCA TCCGTTTA
YerEn_NC_008800.1 ATTTGATA ATCCCAAA ATGCACAT GATGCCCG CGCTGCAA CTGTTTGG CGCCGGTC GTGAAAAA CGCCTGTA TGCCCTGC CACCTTTT ACCAAAGT GGAAAGTC TGGACTTC GACGATCA CCCCTTTA
ShiDy_NC_007606.1 ATTCGATA ACCCAAAA ATGGACAT GATGCCCA CCCTGCAA CTGTTCGG CACAGGGC GCGAAAAG CGCATCTA TGCGGTGC CGCCGTTT ACCCGCGT GGAAAGTC TCGATTTC GACGATCA CCCGTTCA
PseAe_NC_022360.1 GTTCGACA ACCCCAAG CTGGACAT GAGCCCGG CGCTGATG CTGTTCGG CGCAGGTC GCGAGAAA CGCCTGTA CGCGGTGC CGCCGTTC ACCCGGGT GGCGAGCC TGGACTTC GAGGACCA CCCCTTCG
PseAe_NC_022361.1 GTTCGACA ACCCCAAG CTGGACAT GAACCCGG CGCTGATG CTGTTCGG CGCCGGTC GCGAGAAG CGCCTGTA CGCGGTGC CGCCGTTC ACCCGGGT GGCGAGCC TGGATTTC GAGGACCA CCCCTTCG
EntCl_NC_014121.1 ATTCGACA ACCCGAAA ATGGACAT GATGCCTG CCCTGCAG CTGTTCGG TGCCGGGC GCGAAAAG CGCATCTA CGCCGTCC CGCCCTAC ACCCGCGT GGAGAGTC TCGATTTC GATGACCA CCCGTTCA
PseSy_NC_004578.1 ATTCGACA ACCCCAAG CTGCACAT GAGCCCGG CGCTGATG CTGTTCGG TGCCGGAC GCGAAAAA CGCTTGTA CGCCGTGC CGCCGTTC ACCCAGGT GGTGAGCC TAGATTTT GAAGACCA TCCGTTTG
PseAe_NC_022594.1 GTTCGACA ACCCCAAG CTGGACAT GAACCCGG CGCTGATG CTGTTCGG CGCCGGTC GCGAGAAG CGCCTGTA CGCGGTGC CGCCGTTC ACCCGGGT GGCGAGCC TGGATTTC GAGGACCA CCCCTTCG
PseAe_NC_022591.1 GTTCGACA ACCCCAAG CTGGACAT GAACCCGG CGCTGATG CTGTTCGG CGCCGGTC GCGAGAAG CGCCTGTA CGCGGTGC CGCCGTTC ACCCGGGT GGCGAGCC TGGATTTC GAGGACCA CCCCTTCG
VibNi_NC_022528.1 ATTCGACA ACCCGAAA ATGGATCG CATGTCGG CACTGCAA CTGTTCGG AGCCGGTC GGGAAAAG CGCATTTA TGCCATCC CTCCATTT ACCGATGT AAAAAGCC TCGATTTC GAAGACCA CCCATTTG
EscCo_NC_011751.1 ATTCGATA ACCCAAAA ATGGACAT GATGCCCG CCCTGCAG CTGTTCGG CGCAGGAC GCGAGAAG CGCATCTA TGCGGTGC CGCCGTTT ACCCGCGT GGAAAGCC TCGATTTC GACGATCA CCCGTTCA
EscCo_NC_011750.1 ATTCGATA ACCCGAAA ATGGACAT GATGCCCG CCCTGCAG CTGTTCGG CGCAGGGC GCGAGAAG CGCATCTA TGCGGTGC CGCCGTTT ACCCGCGT GGAAAGTC TCGATTTC GACGATCA CCCGTTCA

                  769
EscCo_NC_000913.3 CCGTTCAG CAGTGGGA TGAGCCAT GCGCCATC TGCGGATC GACCCACA G---CTAT CTTGATGA AGTGGTGC TGGATGAC GCCGGAAA CCGCATGT TTGTCTGC TCCGATAC CGATTATT GCCGCCAA
YerPe_NC_003143.1 GCGTACAA CAGTGGGA TGACCCCT GCGCACTG TGTGGTTC GCGCCACA G---TTAT CTTGATGA AGTGGTGA TGGATGAC CAAGGTAG CCGCATGT TTGTTTGC TCGGATAC TGATTTTT GCCAGCAG
YerEn_NC_008800.1 GCGTGCAG CAATGGAA TGAACCCT GTGCTTTG TGCGGCTC ACGCCACA G---TTAT CTCGATGA GGTGGTGA TGGACGAT AAGGGCAG CCGGATGT TTGTTTGT TCGGATAC TGATTTTT GCCAGCAA
ShiDy_NC_007606.1 CCGTTCAG CAGTGGGA TGAGCCAT GCGCCATC TGCGGATC GACCCACA G---CTAT CTCGACGA AGTGGTGC TGGATGAC GCCGGAAA CCGCATGT TTGTCTGC TCCGATAC CGATTATT GCCGCCAA
PseAe_NC_022360.1 AAGTCCAG CGCTGGGA AGAAAGCT GCGCCTTC TGCGGC-- -AGCCACG AGTCCTAC CTGGACGA ACTGATCG TCGACGAC CGTGGCGG CAAGCGCT TCGTCTGT TCCGACAC CGATTACT GCACGCAG
PseAe_NC_022361.1 AGGTTCAG CGCTGGGA GGAAAGCT GCGCCTTC TGCGGC-- -AGCCACG AGTCCTAC CTGGACGA ACTGATCG TCGACGAC CGTGGCGG CAAGCGCT TCGTCTGT TCCGACAC CGATTACT GCACGCAG
EntCl_NC_014121.1 CGGTGCAG GAGTGGGA CGAGCCGT GCGCCATC TGCGGCTC GAAACACA G---CTAT CTGGATGA AGTGGTGC TGGATGAT ACGGGCAA ACGGATGT TTGTCTGC TCCGACAC CGATTACT GCCGCCAA
PseSy_NC_004578.1 AAGTGCAG CGCTGGGA ACAGTGCT GCGCCATC TGCGGC-- -AGCCCTG ATTCGTTT CTCGACGA ACTGATTC TCGATGAC GCCGGAAC ACAGAGTT TTGTCTGT TCCGACAC CGATTACT GCGCCCAG
PseAe_NC_022594.1 AGGTTCAG CGCTGGGA GGAAAGCT GCGCCTTC TGCGGC-- -AGCCACG AGTCCTAC CTGGACGA ACTGATCG TCGACGAC CGTGGCGG CAAGCGCT TCGTCTGT TCCGACAC CGATTACT GCACGCAG
PseAe_NC_022591.1 AGGTTCAG CGCTGGGA GGAAAGCT GCGCCTTC TGCGGC-- -AGCCACG AGTCCTAC CTGGACGA ACTGATCG TCGACGAC CGTGGCGG CAAGCGCT TCGTCTGT TCCGACAC CGATTACT GCACGCAG
VibNi_NC_022528.1 AAGTGCAA CGCTGGGA AGAGCCGT GTGCCCTT TGTGGGGC AACGCATA C---CTTC CTTGACGA AGTAGTGA CCGATGAC CAGGGCAG CCGAATGT ACGTTTGT TCCGATAC CGATTATT GCGCTGAG
EscCo_NC_011751.1 CCGTTCAG CAGTGGGA TGAGCCAT GCGCCATC TGCGGATC GACCCACA G---CTAT CTCGACGA AGTGGTGC TGGATGAC GCCGGAAA CCGCATGT TTGTCTGC TCCGATAC CGATTATT GCCGCCAA
EscCo_NC_011750.1 CCGTTCAG CAGTGGGA TGAGCCAT GCGCCATC TGCGGATC GACCCACA G---CTAT CTCGACGA AGTGGTGC TGGATGAC GCCGGAAA CCGCATGT TTGTCTGC TCCGATAC CGATTATT GCCGCCAA

                  897
EscCo_NC_000913.3 CAG----- -------- -----AGC GAGGCAA- AAAACCAA -------- ------TG A
YerPe_NC_003143.1 CAACTGGC TCAAGCCA GCGCACAA GAAGCCAC ATCCCCAA CCGAGGTA CCTGTCTG A
YerEn_NC_008800.1 CAACTGGC TCAGGCCG CCACACAA GAGGTGA- ATATTCAA -------- ------TG A
ShiDy_NC_007606.1 CAG----- -------- -----AGC GAGGCGA- AAAGCCAA -------- ------TG A
PseAe_NC_022360.1 CGGCGTGC CTGCCAGG AAGAAGGG GAGG---- ACGCGCAA -------- ------TG A
PseAe_NC_022361.1 CGGCGTGC CCGCCAGG AAGAAGGG GAGG---- ACGCGCAA -------- ------TG A
EntCl_NC_014121.1 CAG----- -------- -----AGC GAGGCGA- ACAGCCAA -------- ------TG A
PseSy_NC_004578.1 CGC----- -------G TCAAACAG CAGGAGA- ACGCCCGA -------- ------TG A
PseAe_NC_022594.1 CGGCGTGC CCGCCAGG AAGAAGGG GAGG---- ACGCGCAA -------- ------TG A
PseAe_NC_022591.1 CGGCGTGC CCGCCAGG AAGAAGGG GAGG---- ACGCGCAA -------- ------TG A
VibNi_NC_022528.1 CAAC---- -------- -------- -AGGAGAA ATTGTCAT -------- ----GTTA A
EscCo_NC_011751.1 CAG----- -------- -----AGC GAGGCAA- AAAACCAA -------- ------TG A
EscCo_NC_011750.1 CAG----- -------- -----AGC GAGGCAA- AAAACCAA -------- ------TG A
```
