# Supplementary figures and images for: Glyphosate Shapes a Dinoflagellate-Associated Bacterial Community While Supporting Algal Growth as Sole Phosphorus Source
Source: Front Microbiol. 2017 Dec 19;8:2530. doi: 10.3389/fmicb.2017.02530 (PMC5742145; doi:10.3389/fmicb.2017.02530)

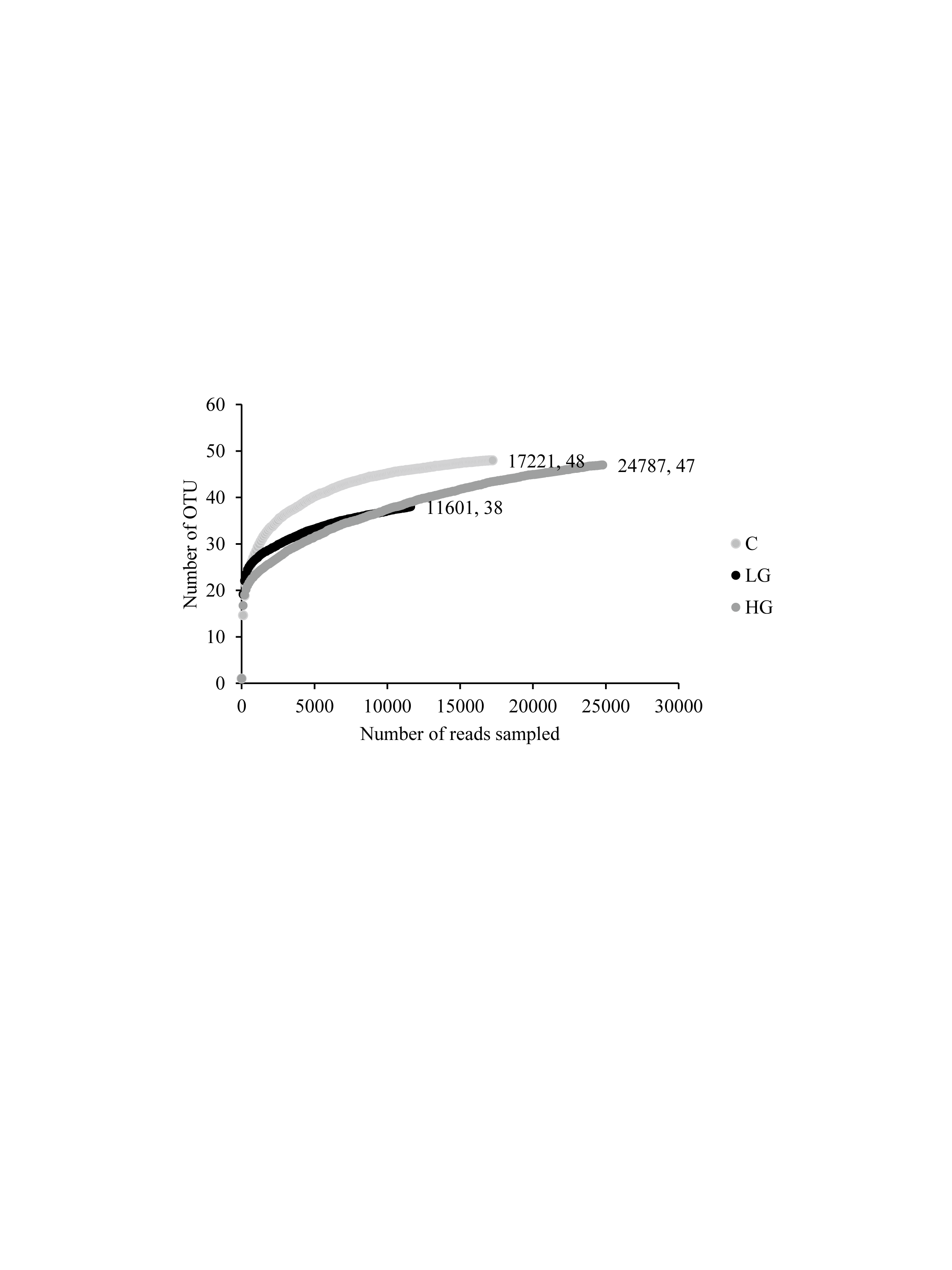

Supplement: Figure S2 — Rarefaction curves for total bacterial communities in the three sampling groups, C, LG, and HG at 3% difference threshold. [file Image2.TIF]

Melt curve for *PhnJ*:

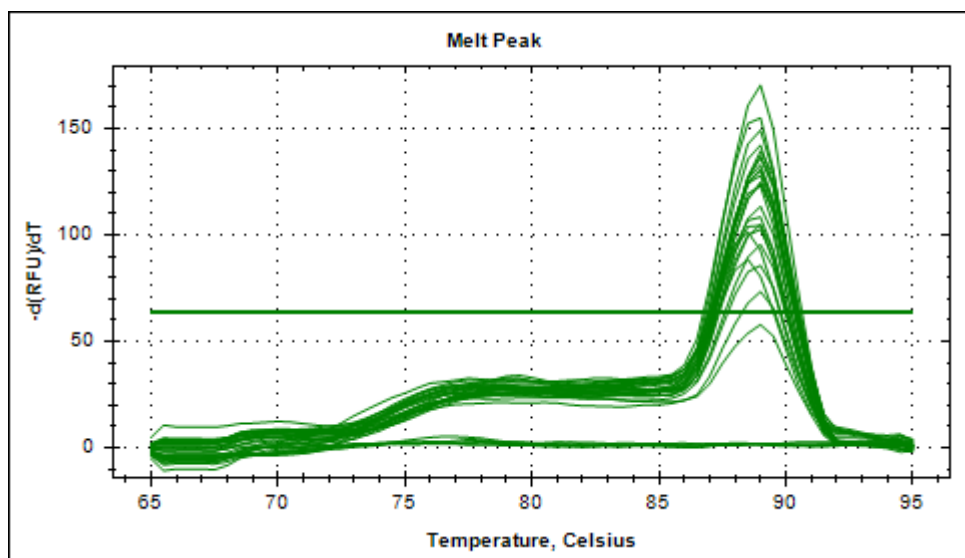

Efficiency curve for *PhnJ*:

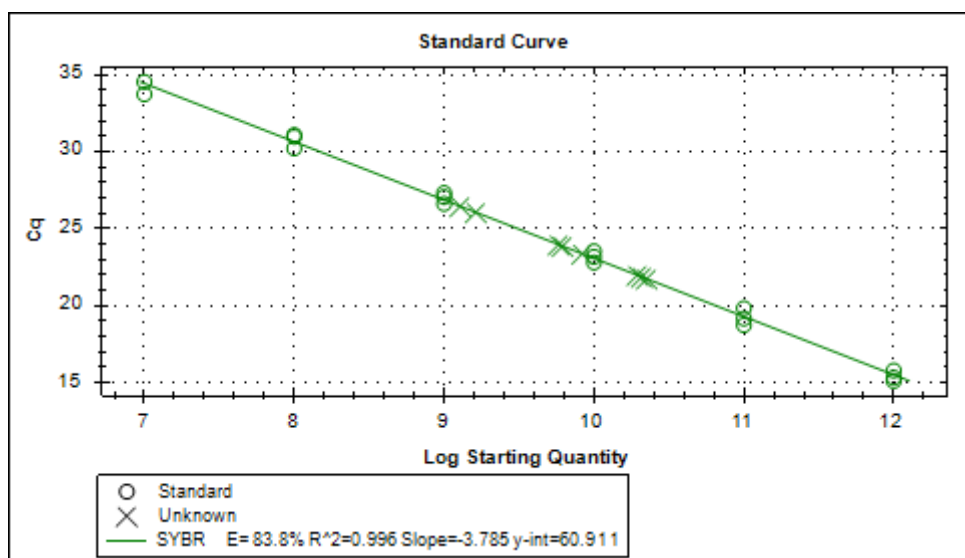

Supplement: Figure S4 — Melting curve and efficiency curve from the qPCR for phnJ gene. [file Image4.PDF]
