# Supplementary material for: Glyphosate Shapes a Dinoflagellate-Associated Bacterial Community While Supporting Algal Growth as Sole Phosphorus Source
Source: Front Microbiol. 2017 Dec 19;8:2530. doi: 10.3389/fmicb.2017.02530 (PMC5742145; doi:10.3389/fmicb.2017.02530)
Supplement: Figure S3 — Cloning sequences from the degenerate primers for phnJ gene. [file Image3.PDF]

>1
TGCATTACTCCGTATTCGTCAAGCGCGTGCATCTTGCGGGTCTCGGTCTCGCGCGGCTCCAGCCAGCGCAGCGGCTCGG
GGATCGGCACCTGGTAGACCAGGATCTGATCCTCGGTCAGCGGCGTCTCCGGGATCCGGTGCCGGGTCTGGATCACCGA
CGCCTCCAGCGTCTTCTCCGTCGTCGCCACGCCGGCGGTCTTGGCGAAGAAGCTGCGGATGTTGACCGCGTTGGTGGTG
TCGTCCGCCCCCTGGTCGATGACCTTAAGGGTGTCGTCCGGGCCGATGACGGCGGCGGTCACCTGAATACCACCGGTTC
CCAACCGTAAGGCATCGGCAT

>2
TGCATTACTCCGTATTCTTCAAGCGCGTGCATCTTGCGGGTCTCGATCTCGGACGGCTCCAGGAACCGCAGAGGTTCCG
GGATCGGCACCTGGTAGACCAGCACCTGATCCTTGGTCAGCGGCTCTTCGGGGATGCGGTGGCGGGTCTGGATGACGCT
GGCCTGCGCGGTTTCCTCGGTGGTCTCGACCCCGGCAGTGCGTTCAAAGAACTTGCGGATGGACACGGCGTTGGTGGTG
TCATCGGCGCCCTGATCGATGACCTTGAGCCGGTCCTCCGGCGTCAGCGTCGCGGCCGATACCTGCATGCCACCGGTGC
CCAGCCATACGGCAGCGGCAT

>3
TGCATTACTCCGTATTCTTCTAGCGCGTGCATCTTGCGGGTCTCGATCTCGGACGGCTCCAGGAACCGCAGAGGTTCCG
GGATCGGCACCTGGTAGACCAGCACCTGATCCTTGGTCAGCGGCTCTTCGGGGATGCGGTGGCGGGTCTGGATGACGCT
GGCCTGCGCGGTTTCCTCGGTGGTCTCGACCCCGGCAGTGCGTTCAAAGAACTTGCGGATGGACACGGCGTTGGTGGTG
TCATCGGCGCCCTGATCGATGACCTTGAGCCGGTCCTCCGGCGTCAGCGTCGCGGCCGATACCTGCATACCACCGGTGC
CCAACCGTAAGGCAGCGGCAT

>4
TGCATTACACCGTATTCTTCAAGCGCGTGCATCTTGCGGGTCTCGATCTCGGACGGCTCCAGGAACCGCAGAGGTTCCG
GGATCGGCACCTGGTGGACCAGCACCTGATCCTTGGTCAGCGGCTCTTCGGGGATGCGATGGCGGGTCTGGATGACGCT
GGCCTGCGCGGTTTCCTCGGTGGTCTCGACCCCGGCAGTGCGTTCAAAGAACTTGCGGATGGACACGGCGTTGGTGGTG
TCATCGGCGCCCTGATCGATGACCTTGAGCCGGTCCTCCGGCGTCAGCGTCGCGGCCGATACCTGCATACCACCGGTGC
CCAACCATAAGGCAGCGGCAT

>5
TGCATTACTCCGTAGTCTTCTAGCGCATGCATCTTGCGGGTCTCGGTCTCGCGCGGCTCCAGCCAGCGCAGCGGCTCGG
GGATCGGCACCTGGTAGACCAGGATCTGATCCTCGGTCAGCGGCGTCTCCGGGATCCGGTGCCGGGTCTGGATCACCGA
CGCCTCCACCGTCTTCTCCGTCGTCGCCACGCCGGCGGTCTTGGCGAAGAAGCTGCGGATGTTGACCGCGTTGGTGGTG
TCGTCCGCCCCCTGGTCGATGACCTTAAGGGTGTCGTCCGGGCCGATGACGGCGGCGGTCACCTGAATACCGCCGGTTC
CCAACCGTACGGCAGTGGCAT

>6
TGCATAACTCCGTACTCGTCTAGCGCGTGCATCTTGCGGGTCTCGATCTCGGACGGCTCCAGGAACCGCAGAGGTTCCG
GGATCGGCACCTGGTAGACCAGCACCTGATCCTTGGTCAGCGGCTCTTCGGGGATGCGGTGGCGGGTCTGGATGACGCT
GGCCTGCGCGGTTTCCTCGGTGGTCTCGACCCCGGCAGTGCGTTCAAAGAACTTGCGGATGGACACGGCGTTGGTGGTG
TCATCGGCGCCCTGATCGATGACCTTGAGCCGGTCCTCCGGCGTCAGCGTCGCGGCCGATACCTGCATGCCGCCGGTTC
CCAACCGTAGGGCATCGGCAT

>7
TGCATTACTCCGTATTCGTCTAGCGCGTGCATCTTGCGGGTCTCGATCTCGGACGGCTCCAGGAACCGCAGAGGTTCCG

GGATCGGCACCTGGTAGACCAGCACCTGATCCTTGGTCAGCGGCTCTTCGGGGATGCGGTGGCGGGTCTGGATGACGCT
GGCCTGCGCGGTTTCCTCGGTGGTCTCGACCCCGGCAGTGCGTTCAAAGAACTTGCGGATGGACACGGCGTTGGTGGTG
TCATCGGCGCCCTGATCGATGACCTTGAGCCGGTCCTCCGGCGTCAGCGTCGCGGCCGATACCTGCATACCACCGGTTC
CCAACCATACGGCAGTGGCAT

>8
TGCATTACTCCGTATTCTTCTAGCGCGTGCATCTTGCGGGTCTCGATCTCGGACGGCTCCAGGAACCGCAGAGGTTCCG
GGATCGGCACCTGGTAGACCAGCACCTGATCCTTGGTCAGCGGCTCTTCGGGGATGCGGTGGCGGGTCTGGATGACGCT
GGCCTGCGCGGTTTCCTCGGTGGTCTCGACCCCGGCAGCGCGTTCAAAGAACTTGCGGATGGACACGGCGTTGGTGGTG
TCATCGGCGCCCTGATCGATGACCTTGAGCCGGTCCTCCGGCGTCAGCGTCGCGGCCGATACCTGCATGCCACCGGTGC
CCAACCATACGGCATTGGCAT
